# Application of ultrasound and methylation omics detection in the diagnosis of endometrial cancer

**DOI:** 10.3389/fonc.2026.1803441

**Published:** 2026-06-03

**Authors:** Dongmei Liu, Wenzhe Hu, Nan Luo, Yangzheng Xia, Fang Liu, Zhenzhen Cheng, Binyu Zheng, Yong Liu, Jirun Peng

**Affiliations:** 1 Department of Ultrasound, Beijing Shijitan Hospital, Capital Medical University, Beijing, China; 2 Department of Surgery, Beijing Shijitan Hospital, Capital Medical University, Beijing, China; 3 Capital Center for Children’s Health, Capital Medical University, Capital Institute of Pediatrics, Beijing, China

**Keywords:** cervical scrapings, diagnosis, DNA methylation analysis, endometrial cancer, ultrasound imaging

## Abstract

**Background:**

Endometrial cancer (EC) is a common malignant gynecological tumor with a rising incidence globally. This study aimed to integrate DNA methylation analysis of cervical scrapings with transvaginal ultrasound (TVUS) imaging to develop a more effective non-invasive diagnostic strategy for EC.

**Methods:**

Methylated cytosine-guanine dinucleotide (CpG) tandem amplification and sequencing was used for genome-wide methylation profiling on EC tissues, adjacent normal tissues, and cervical scrapings from 20 EC patients and 20 benign endometrial thickening controls. Samples were randomly divided training and validation cohorts at a ratio of 6:4. Sequencing depth was 30× with coverage ≥90%, and repeatability was validated (intraclass correlation coefficient ≥0.85). BWA-Meth was used for alignment and the β-value method for methylation level calculation. Strict quality control was applied (DNA concentration ≥20 ng/μL, OD260/280 = 1.8~2.0) with preset sample criteria (≥5000 cells, ≥40% cell proportion, ≥20 mg weight). TVUS parameters were correlated with methylation findings. Four diagnostic strategies were evaluated: methylation biomarkers alone, TVUS alone, direct combination, and sequential approach (TVUS followed by methylation testing).

**Results:**

Significant differences were observed in Adler blood flow grade and endometrial-myometrial border irregularity between EC and control groups (*P* < 0.05). Methylation analysis identified 2,671 differentially methylated CpG sites corresponding to 745 differentially methylated genes (DMGs). Three core methylation biomarkers (BAHCC1-8721-2-1, SHANK3-16286-1-5, FHL1-26624-1-3) were screened in the training set, with single-gene area under the curve (AUC) of 0.804, 0.782, and 0.844, respectively. Combined methylation biomarkers achieved an AUC of 0.922 (sensitivity 85.0%, specificity 90.0%). TVUS alone had an AUC of 0.725; direct combination yielded an AUC of 0.900. The sequential approach showed the highest performance with an AUC of 0.950 (sensitivity 90.0%, specificity 95.0%). Delong test confirmed significant differences between the sequential strategy and TVUS alone or direct combination (*P* < 0.05).

**Conclusion:**

Cervical scraping-based methylation analysis may serve a useful non-invasive tool for EC detection. The sequential combination of TVUS and methylation testing appears to improve diagnostic accuracy, suggesting a potential strategy for early EC screening. This is a single-center exploratory study with a small sample size, and the results need to be validated in large-scale, multi-center independent cohorts.

## Introduction

The incidence of endometrial carcinoma (EC) is continuously rising, making it the most common gynecological malignancy in economically developed regions of China (1–3). Transvaginal ultrasonography (TVUS) is the first-line imaging modality for EC screening, however, its diagnostic accuracy for early-stage EC remains suboptimal (4). This leads to unnecessary hysteroscopy or endometrial biopsy in low-risk patients and delayed treatment due to missed diagnosis in early-stage EC patients. Therefore, a more accurate and efficient adjunctive method for TVUS screening is urgently needed.

Epigenetic abnormalities, particularly DNA methylation alterations, play a crucial role in the initiation and progression of EC (5, 6). Aberrant hypermethylation of cytosine-guanine dinucleotide (CpG) sites in gene promoter regions can induce transcriptional silencing of tumor suppressor genes (7–9), which precedes histological morphological changes and provides potential molecular targets for early EC intervention. Methylated biomarkers detected in vaginal secretions from urine or tampons have shown diagnostic potential, but their clinical application is limited by low DNA yield and poor regional scalability (10, 11). Elevated methylation levels of ADCYAP1 and HAND2 in endometrial biopsies are associated with EC risk (12), however, biopsies are invasive and unsuitable for large-scale screening.

Cervical scrapings, a classic specimen for cervical cancer screening, have distinct advantages for EC screening due to their non-invasive collection and easy procurement. Compared with urine and tampons, cervical scrapings provide higher-quality tumor DNA and avoid the invasiveness of tissue biopsies, making them more suitable for young women. Previous studies have reported that methylation levels of HIST1H4F and RASSF1A in cervical scrapings are positively associated with EC pathological staging (13–15), indicating the potential of epigenetic testing of cervical scrapings for early EC screening.

This study is not the first to report DNA methylation analysis of cervical scrapings for EC detection, but it achieves three novel contributions compared with previous studies: (1) Identifying three novel methylation biomarkers (BAHCC1, SHANK3, FHL1) specifically suitable for cervical scraping detection of EC; (2) Establishing a rigorous biomarker screening workflow based on the division of training and validation sets to ensure objectivity and reliability; (3) Proposing the first sequential combination strategy of TVUS and cervical scraping methylation detection that simulates the actual clinical screening workflow.

This study used methylated CpG tandem amplification and sequencing (MCTA-Seq) method to detect DNA methylation biomarkers in cervical scrapings and evaluate their diagnostic efficacy alone and in combination with TVUS. By integrating imaging and epigenetic data, we aimed to construct a novel non-invasive screening system for early EC diagnosis, which may improve the early diagnosis rate of EC and protect the health of women of reproductive age.

## Methods

### Study design and participants

This retrospective cohort study was conducted on EC patients and age-matched patients with benign endometrial thickening (controls) who were treated at Beijing Shijitan Hospital, Capital Medical University. The control group consisted of patients with histopathologically confirmed benign endometrial thickening. This selection reflects clinical practice, as TVUS screening primarily targets high-risk individuals with endometrial thickening, allowing for an objective evaluation of the diagnostic value of the proposed strategy. Inclusion criteria for the EC group were: (1) surgical treatment performed at our hospital; (2) histopathological confirmation of EC; (3) availability of complete TVUS data and diagnostic images; (4) a cervical scraping methylation detection positive rate >50% (a sample quality control standard to ensure effective detection of tumor-related methylation signals, not a biomarker screening criterion).

Exclusion criteria for the EC group included: (1) concurrent malignancies (such as colorectal, gastric and lung cancer); (2) pregnancy or lactation; (3) preoperative radiotherapy or chemotherapy.

Inclusion criteria for the control group were: (1) age ≥ 18 years; (2) histopathologically confirmed benign endometrial thickening; (3) availability of complete TVUS data and diagnostic images.

Exclusion criteria for the control group included: (1) precancerous lesions, such as endometrial atypical hyperplasia; (2) genital organic diseases (such as uterine fibroids and ovarian cancer); (3) pregnancy or lactation; (4) a history of malignant tumors.

The study was approved by the Ethics Committee of Beijing Shijitan Hospital (Approval Number: IIT2024-117-002). All participants provided written informed consent, and personal information was de-identified to ensure privacy.

### Sample collection

Cervical scrapings were obtained from 20 EC patients, along with EC tissue samples and adjacent normal tissues (20 pairs), and from 20 control participants with benign endometrial thickening. All participants underwent TVUS examination, and cervical scrapings from EC patients were collected preoperatively.

Samples containing fewer than 5,000 cells, a cell proportion below 40%, or weighting less than 20 mg were excluded. These criteria were pre-established based on the minimum sample requirements for MCTA-Seq technology and pre-experiment results, ensuring stable and reliable sequencing data (16). All eligible samples were randomly allocated to training (12 EC patients + 12 controls) and validation (8 EC patients + 8 controls) groups at a 6:4 ratio using a random number table, maintaining balanced baseline data between the two groups.

### DNA extraction and MCTA-Seq library construction

Genomic DNA was extracted from endometrial tissue and cervical scraping cells using the DNeasy Blood and Tissue Kit (Qiagen, 69504) according to the manufacturer’s instructions. DNA concentration and purity were assessed using a NanoDrop 2000, with quality control criteria set as DNA concentration ≥20 ng/μL and OD260/280 between 1.8 and 2.0. Samples failing to meet these criteria were excluded. Genomic DNA was then subjected to bisulfite conversion using the EZ DNA Methylation-Gold™ Kit (Zymo Research, D5006) following the manufacturer’s protocol. Amplification and purification of the MCTA-Seq library were performed as previously reported (17), with optimized experimental parameters. Each 25 μL PCR reaction system contained 0.5 μmol/L of each primer A, B, C, and D, 50 ng of sample DNA, 2.5 μL 10×Ex Taq buffer (Takara, RR006B), 2 μL of 2.5 mM dNTPs (Takara, RR006B), 0.25 μL of 5 U/μL HS Ex Taq enzyme (Takara, RR006B), 16.25 μL of nuclease-free water (Ambion, AM9932), and 2.5 μL of Klenow fragment (3’-5’ exonuclease, NEB, M0212M). PCR cycling conditions were: 94°C pre-denaturation for 5 min, 35 cycles of 94°C denaturation for 30 s, 58°C annealing for 30 s, 72°C extension for 30 s, followed by a final extension at 72°C for 10 min. For library purification, the amplified products were mixed with primers QP-1 (5’-AATGATACGGCGACCACCGA-3’) and QP-2 (5’-CAAGCAGAAGACGGCATACGA-3’) for 1–2 cycles of secondary amplification. Products were then purified using AMPure XP magnetic beads, selecting fragments of 200–400 bp.

Repeatability validation: Three EC tissue samples and three cervical scraping samples were randomly selected for three independent MCTA-Seq assays. The intraclass correlation coefficient (ICC) of methylation levels was calculated, with ICC≥0.85 indicating good repeatability of the technique.

### Function and pathway annotation of differential methylated genes

Based on a classical Bayes framework, batch effects in endometrial tissue and cervical scraping samples were corrected using the ComBat function. Differential methylation between cancerous and normal tissue was assessed using a threshold of *P* < 0.05 and |log_2_ fold change (FC)| > 1, where log_2_ FC = log_2_ (methylation level in the target group/methylation level in the control group)). Volcano plots and principal component analysis (PCA) were constructed to visualize the distribution of differential methylated genes (DMGs). Functional annotation of DMGs was performed using Gene Ontology (GO) and Kyoto Encyclopedia of Genes and Genomes (KEGG) pathway analyses via the R package clusterProfiler (version 4.0.5).

### Screening of methylation biomarkers

Biomarker screening was conducted exclusively on the training set to avoid overfitting, with the validation set reserved for independent efficacy verification. For genome-wide CpG sites (excluding the Y chromosome), the following multi-dimensional criteria were applied based on previous studies (18, 19) and pre-experiment results: (1) mean methylation level in cancer tissue significantly higher than in normal tissue (*P* < 0.05, |log_2_ FC| >1); (2) median methylation level in cancer tissue > 0; (3) positive rate in normal tissue < 10%; (4) positive rate in cervical scrapings from EC patients > 50% (sample quality control); (5) positive rate in cervical scrapings from normal controls < 10%. Only CpG sites meeting all criteria were considered potential methylation biomarkers. Optimal cut-off values were determined based on the Youden index from the receiver operator characteristic (ROC) curve of the training set. Finally, biomarkers with area under the curve (AUC)>0.75 in the training set were selected as core methylation biomarkers.

### Assessment of diagnostic performance

The diagnostic performance of four strategies was evaluated: (1) TVUS alone; (2) methylation biomarkers alone; (3) direct combination of TVUS and methylation biomarkers; (4) a sequential approach simulating clinical workflow.

TVUS result classification criteria were as follows: (1) normal: Adler blood flow grade ≤I, with a regular endometrial-myometrial border (EMB); (2) suspicious: Adler blood flow grade II~III, or irregular EMB; (3) unclear: inconsistent blood flow grade and EMB results.

Sequential approach: methylation testing was performed for patients with suspicious or unclear TVUS results. Patients with normal TVUS results were directly classified as negative. Positive methylation results were considered as EC positive, whereas negative methylation results were considered as EC negative.

### Statistics and analysis

Continuous variables were presented as mean ± standard deviation and compared using the independent t-test. Categorical variables were presented as counts and percentage (%) and compared using the chi-square test. ROC curves were plotted to evaluate diagnostic performance, with primary indicators including AUC, sensitivity, specificity, positive predictive value (PPV), negative predictive value (NPV), and their 95% confidence intervals (CI). Delong test was used for the comparison of AUC between different diagnostic strategies (20). Batch effects were corrected using the ComBat function. All statistical analyses were conducted by SPSS 26.0 and R 23.3.0 software, with *P* < 0.05 considered statistically significant.

## Results

### Characteristics of patients and TVUS findings

A total of 40 participants were enrolled, including 20 histopathologically confirmed EC patients and 20 controls with endometrial thickening. The baseline characteristics of the two groups are shown in **Table 1**. The mean age of EC patients was 58.60 ± 11.35 years, and that of controls were 54.55 ± 8.69 years, with no significant difference between the two groups (t=1.24, *P* = 0.23).

**Table 1:** Characteristics of patients and TVUS findings.

Variables	EC cases (n=20)	Control (n=20)	χ^2^/t	*P*
Age (years)	58.60 ± 11.35	54.55 ± 8.69	1.24	0.23
Adler blood flow grade, n (%)			4.91	0.03
≤I	7 (35.0)	14 (70.0)		
II-III	13 (65.0)	6 (30.0)		
Uniform lesion echo, n (%)			1.76	0.19
Yes	5 (25.0)	9 (45.0)		
No	15 (75.0)	11 (55.0)		
Regular EMB, n (%)			12.91	<0.01
Yes	7 (35.0)	18 (90.0)		
No	13 (65.0)	2 (10.0)		
Endometrial thickness (cm)	2.05 ± 1.20	1.67 ± 0.80	1.13	0.27

EC, endometrial cancer; EMB, endometrial-myometrial border; χ^2^, chi-square test; t, independent t-test.

**Figure 1:**
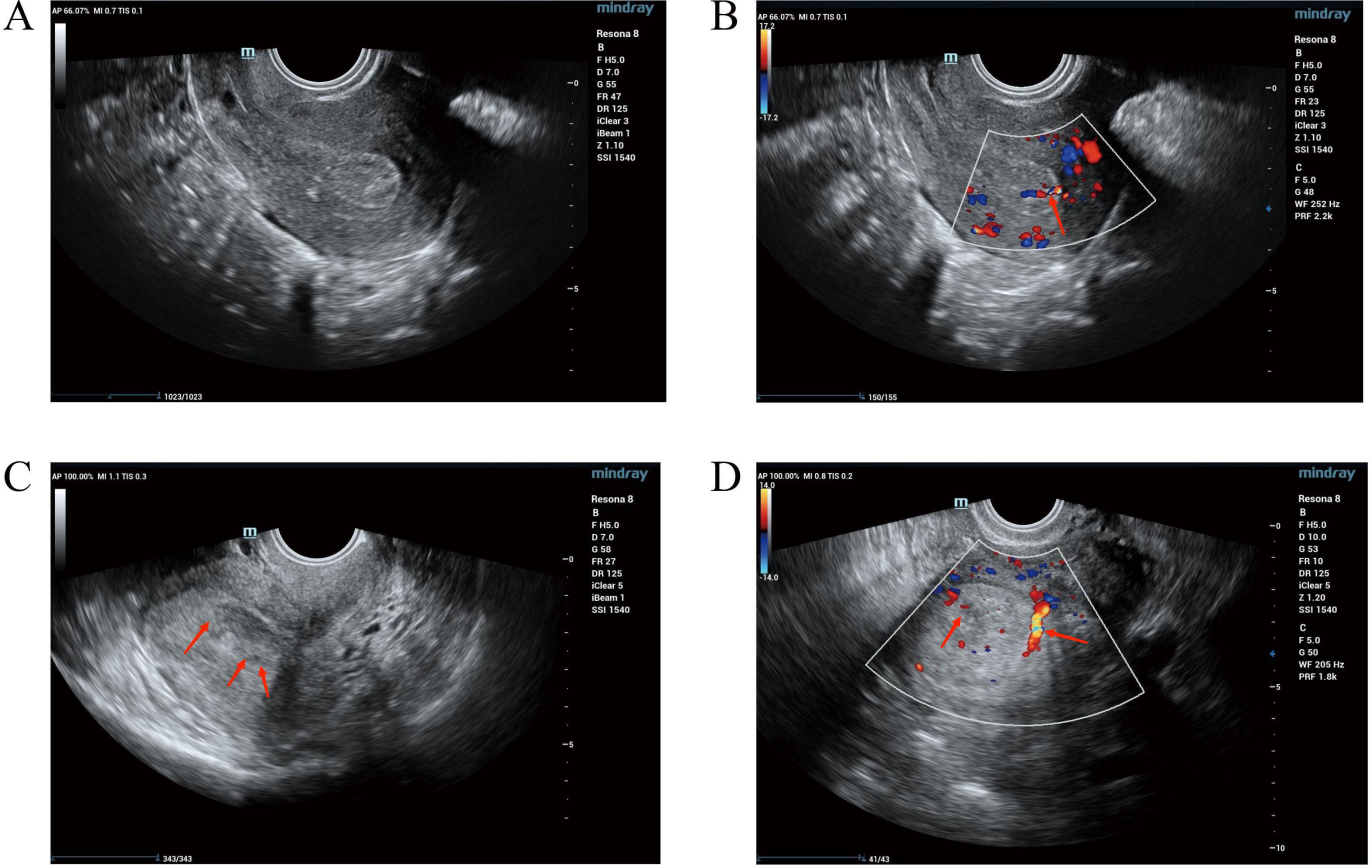
Representative ultrasound images of endometrial lesions. **(A)** Ultrasound image of a patient with endometrial thickening in the control group, showing a clear boundary between the endometrium and myometrium; **(B)** Corresponding color Doppler image of the same patient, demonstrating a strip-like blood vessel with Adler blood flow grade I; **(C)** Ultrasound image of a patient with endometrial carcinoma, showing an indistinct boundary between the endometrium and myometrium of the posterior uterine wall; **(D)** Corresponding color Doppler image of the same endometrial carcinoma patient, showing disordered and abundant blood flow with Adler blood flow grade II.

TVUS findings showed significant differences in Adler blood flow grade and EMB regularity between the EC and the control groups (*P* < 0.05, **Table 1**, **Figure 1**). In the EC group, 65.0% (13/20) of patients had Adler blood flow grade II~III, which was significantly higher than 30.0% (6/20) in the control group (χ^2^=4.91, *P* = 0.03). 65.0% (13/20) of EC patients had irregular EMB, compared with 10.0% (2/20) in the control group (χ^2^=12.91, *P* < 0.01). No significant differences were observed in endometrial thickness and lesion echogenicity between the two groups (*P*>0.05).

### Differential methylomics analysis between EC and normal endometrium

**Figure 2:**
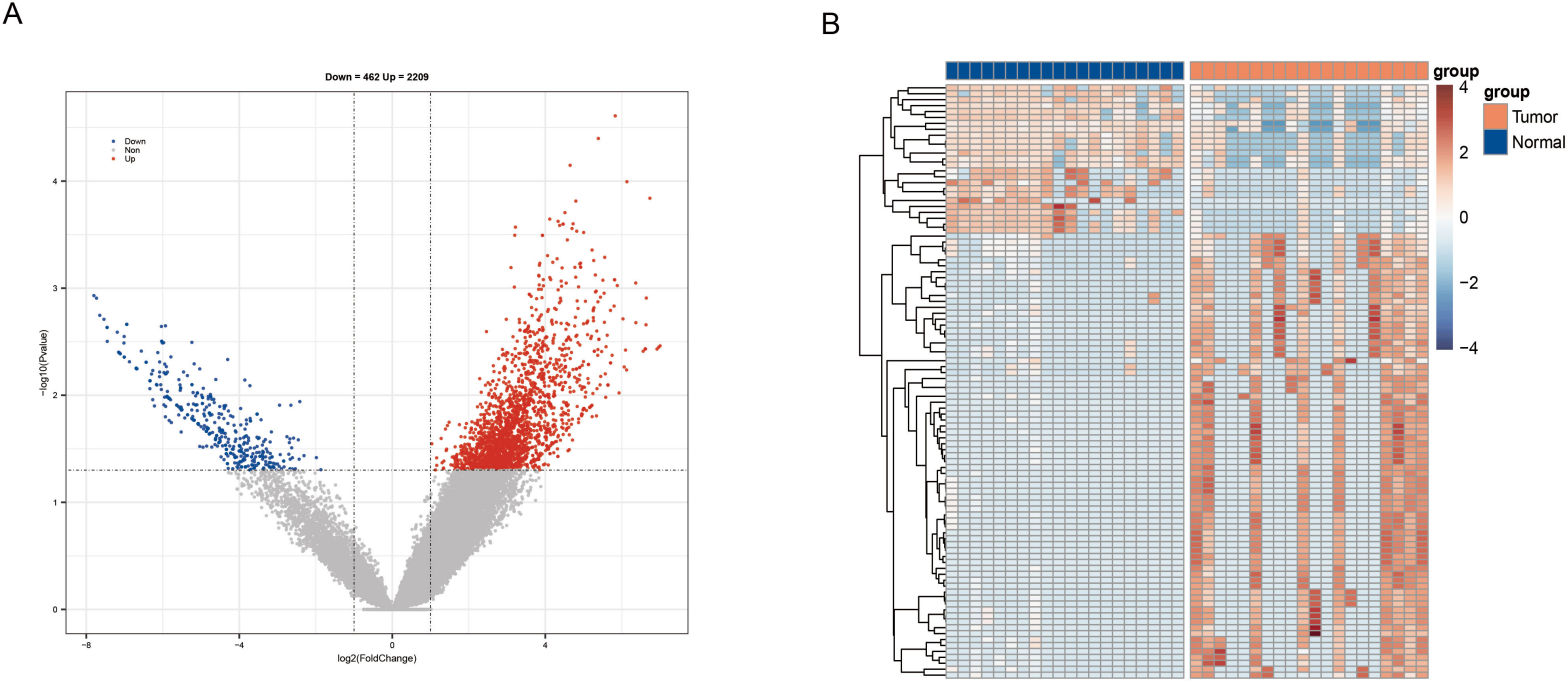
Volcano and heat map plots illustrating DMGs from all tissue samples. **(A)** Red data points denote upregulated DMGs, whereas blue data points indicate downregulated DMGs; **(B)** Each row of the heat map corresponds to one gene, and each column corresponds to one sample. The red and blue colors signify upregulated and downregulated DMGs, respectively.

Genome-wide methylation sequencing of EC tissues and paired normal tissues identified 2,671 differentially methylated CpG sites (*P* < 0.05, |log_2_FC| >1), corresponding to 745 DMGs. Among these, 2,209 sites were hypermethylated and 462 were hypomethylated in EC tissues (**Figure 2A**). The heat map showed that the distribution pattern of differentially methylated sites in cancerous and normal tissues were significantly different, consistent with the sequencing results (**Figure 2B**; all DMGs are labeled in the **Supplementary Material**). Further comparison of cervical scrapings between the EC group and the control group identified an additional 4,647 differentially methylated CpG sites. These results indicated that significant epigenetic alterations occur in both endometrial tissue and cervical cells during EC carcinogenesis, mainly characterized by elevated methylation levels in the promoter regions of most genes.

### Principal component analysis and enrichment analysis

**Figure 3:**
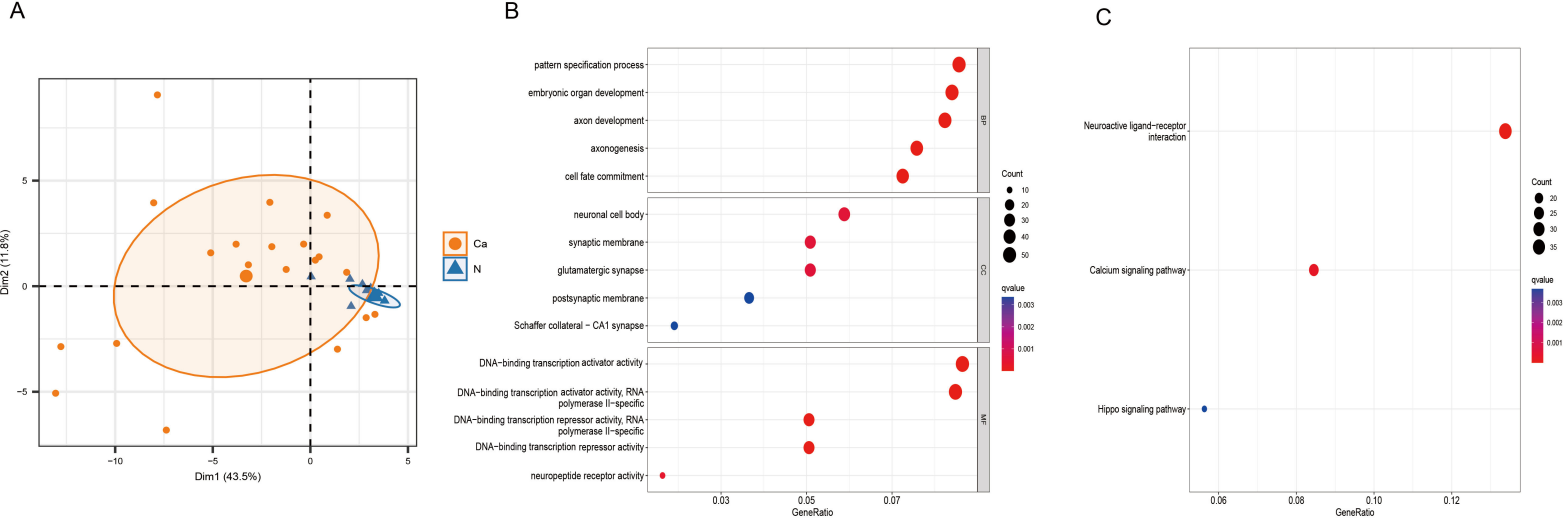
PCA, GO analysis, and KEGG pathway analysis of differentially methylated genes in tissue samples. **(A)** The orange color denotes cancer tissue, while the blue points indicate normal tissue, with a total of 40 data points; **(B)** GO analysis, including biological process, cellular component, and molecular function; **(C)** KEGG analysis, where the size of the points reflects the number of genes and the color depth signifies the significance.

PCA of the 745 tissue-derived DMGs showed a clear separation between cancerous and normal tissue clusters, with only 3 normal tissue samples overlapping with the EC cluster (**Figure 3A**), indicating these DMGs can effectively distinguish EC lesions from normal endometrial tissues.

GO analysis: DMGs were significantly enriched in biological processes related to “pattern specification process”. Cellular component related to “neuronal cell bodies”, and molecular functions related to “DNA-binding transcription activator activity” (**Figure 3B**). These results only reflect the epigenetic characteristics of DMGs and have not been confirmed to have a direct biological association with EC occurrence.

KEGG pathway analysis: DMGs were most significantly enriched in the “neuroactive ligand-receptor interaction” and the “calcium signaling pathway” (**Figure 3C**). Calcium homeostasis imbalance may affect estrogen receptor signaling, and neuroendocrine factor abnormalities may be involved in tumor microenvironment regulation (21). This finding is associated with the hormone-dependent nature of EC and provides a preliminary clue for exploring the mechanism of DNA methylation in EC pathogenesis.

### DNA methylation biomarkers in cervical scrapings

**Figure 4:**
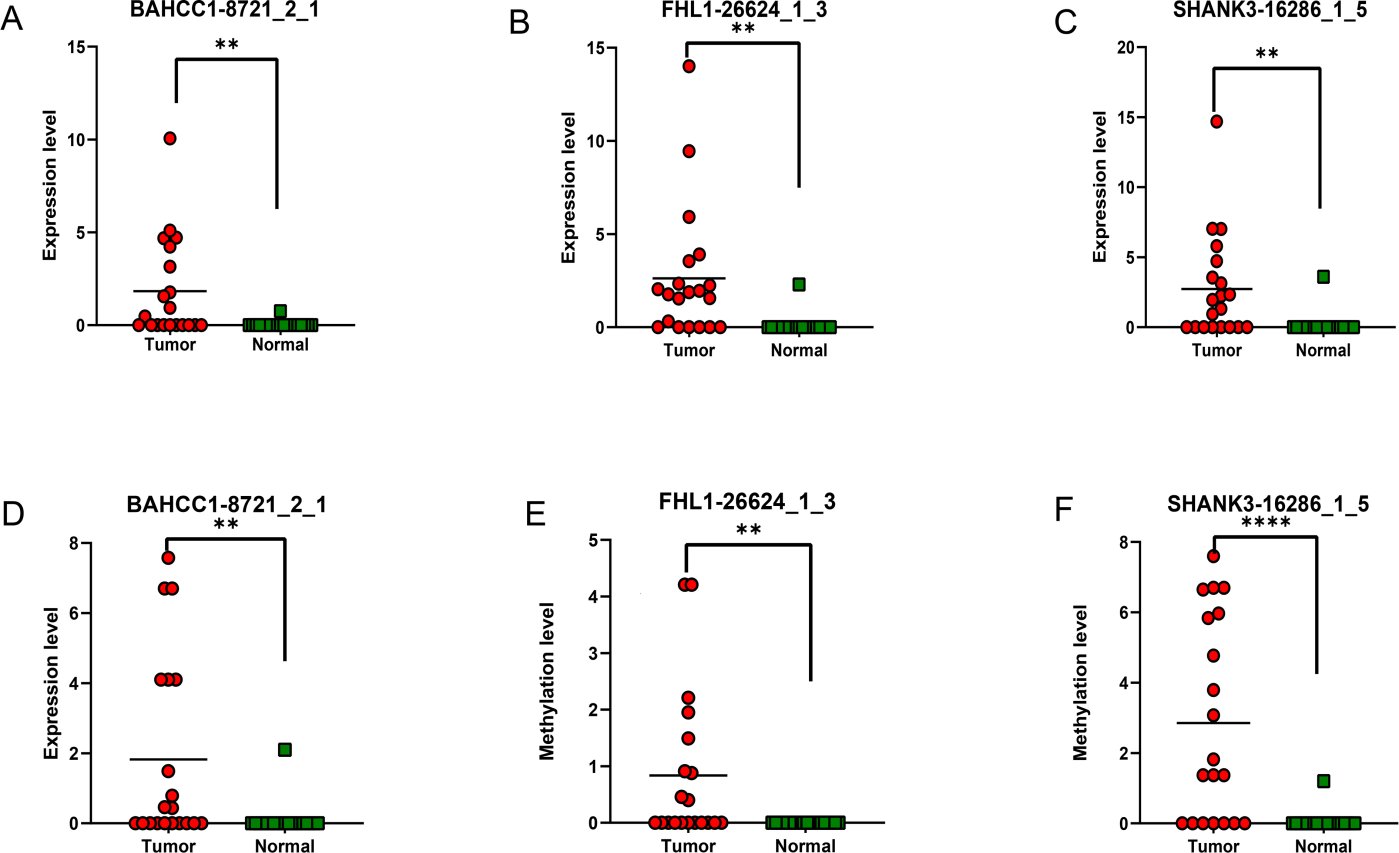
MCTA-Seq was employed to screen methylated DNA biomarkers for EC. **(A–C)** Expression differences of BAHCC1, FHL1, and SHANK3 in tissue samples; **(D–F)** Expression differences of BAHCC1, FHL1, and SHANK3 in cervical scrapings.

Based on the training set data, three core methylation biomarkers were screened out from the genome-wide CpG sites using the multi-dimensional criteria: BAHCC1-8721-2-1 (chromosome 17), SHANK3-16286-1-5 (chromosome 22), and FHL1-26624-1-3 (X chromosome). Validation set results showed that the methylation levels of these three genes were significantly higher in EC tissues than in normal tissues, and their positive rates in cervical scrapings from EC patients were significantly higher than those in the control group (**Figures 4A–F**).

**Figure 5:**
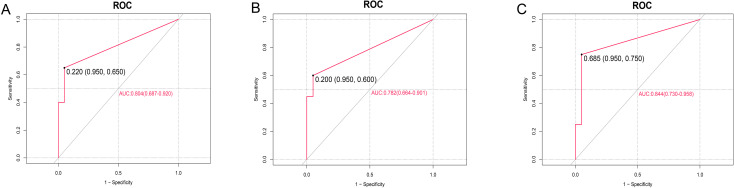
ROC analysis of methylated DNA biomarkers for diagnosing EC. **(A–C)** ROC analysis of BAHCC1, FHL1, and SHANK3 for diagnosing EC.

The three DMGs showed different diagnostic performance when used alone for EC diagnosis (ROC curve values were rechecked and corrected, **Figures 5A–C**): SHANK3 had the best performance with an AUC of 0.844 (95%CI: 0.730~0.958), followed by BAHCC1 with an AUC of 0.804 (95%CI: 0.687~0.920), and FHL1 with an AUC of 0.782 (95%CI: 0.664~0.901).

### Comparison of the diagnostic performance of methylation markers and TVUS

**Figure 6:**
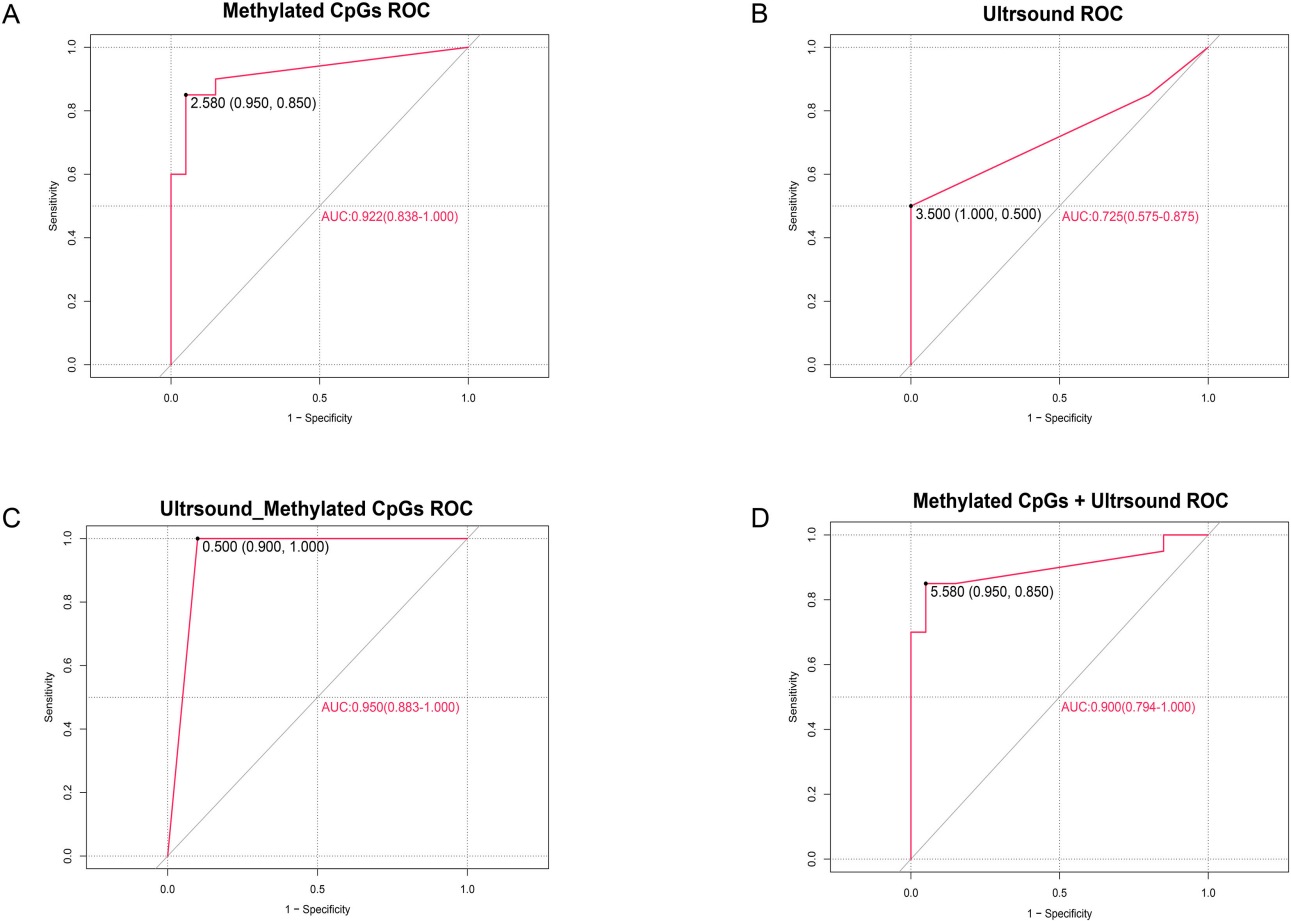
Performance comparison among four diagnostic methods. **(A)** Application of DNA methylation biomarkers alone; **(B)** Application of TVUS parameters alone; **(C)** Sequential application of methylation biomarkers and TVUS; **(D)** Straightforward combination of methylation biomarkers and TVUS.

ROC curve analysis of the four diagnostic strategies (ROC curve values were rechecked and corrected, **Figure 6**) showed that the sequential approach had the highest diagnostic performance with an AUC of 0.950 (95%CI: 0.883~1.000). Methylation biomarkers alone had an AUC of 0.922 (95%CI: 0.838~1.000), followed by the direct combination with an AUC of 0.900 (95%CI: 0.794~1.000). TVUS alone had the lowest performance with an AUC of 0.725 (95%CI: 0.575~0.875).

The detailed diagnostic efficacy indicators of the four strategies are evaluated. Delong test showed that the AUC of the sequential approach was significantly higher than that of TVUS alone (Z = 2.13, *P* = 0.03) and the direct combination (Z = 2.01, *P* = 0.04), with no significant difference between the sequential approach and methylation biomarkers alone (*P*>0.05).

## Discussion

This study established a sequential combination strategy of TVUS and cervical scraping DNA methylation detection for non-invasive diagnosis of EC (AUC = 0.950), superior to TVUS alone, methylation biomarkers alone, and direct combination. This strategy conforms to real clinical workflow, retaining the simplicity and low cost of TVUS while boosting accuracy via methylation testing, showing good translational potential.

TVUS is the first-line imaging modality for EC screening, but its discriminatory power for early EC and benign endometrial thickening is suboptimal (22, 23). Consistent with previous reports, our study found that there were no significant differences in endometrial thickness or echogenicity between EC patients and controls (24, 25). Regarding blood flow signals, early-stage EC typically exhibits low-resistance blood flow. Nevertheless, some benign lesions may also exhibit similar blood flow characteristics (26). Furthermore, ultrasound assessment of blood flow and EMB integrity is inherently subjective, influenced by operator experience and equipment sensitivity. Although our study confirmed significant differences in Adler blood flow grade and EMB irregularity between groups (*P* < 0.05), the diagnostic performance of TVUS alone (AUC = 0.725) underscores its insufficiency for reliably distinguishing early malignant lesions from benign mimics.

DNA methylation abnormalities precede morphological changes and can capture early molecular events of EC (27). The 745 DMGs identified in this study were mainly enriched in DNA transcription activation and calcium signaling pathways, which are related to the hormone-dependent feature of EC, providing preliminary epigenetic clues for pathogenesis, though direct biological functions need further verification. Calcium ion homeostasis imbalances can influence estrogen receptor signaling, whereas neuroendocrine factor abnormalities may be implicated in tumor microenvironment regulation (21). This discovery correlates with the hormone-dependent nature of EC, potentially elucidating part of the underlying mechanism of DNA methylation in EC pathogenesis.

Among the numerous DMGs, we identified three core biomarker:BAHCC1-8721-2-1 (chromosome 17), FHL1-26624-1-3 (X chromosome), and SHANK3-16286-1-5 (chromosome 22), with significant diagnostic utility in cervical scrapings. Unlike TVUS, methylation detection, by capturing abnormal methylation signals in cervical exfoliated cells, can identify lesions prior to the occurrence of morphological changes (28). Our data revealed that combining three methylation biomarkers yielded diagnostic performance (AUC = 0.922) markedly superior to TVUS alone (AUC = 0.725), indicating their potential values in diagnosing EC. A key innovation of this study was that the sequential strategy enhanced the AUC from 0.90 to 0.95, indicating its potential applicability in primary care settings. The clinical advantages of the sequential strategy included simple operation, low cost for initial TVUS screening, reduced unnecessary biopsies, suitable for primary care settings. And its potential real-world application scenarios included EC early screening for high-risk groups with endometrial thickening.

BAHCC1 is situated on autosome 17, and the physiological function is closely associated with epigenetic regulation. BAHCC1 recognizes the trimethylation modification of histone H3 lysine 27 (H3K27me3) via its conserved BAH domain and mediates gene silencing as a reader (29). Study has demonstrated that BAHCC1 is abnormally overexpressed in acute leukemia (30). By binding to gene regions marked by H3K27me3, it inhibits the expression of tumor suppressor genes and promoting cancer cell proliferation and invasion (30). Our finding of BAHCC1 hypermethylation in EC tissues suggests a potential oncogenic role through epigenetic regulation.

SHANK3 is located on autosome 22 and is predominantly expressed in the postsynaptic density of excitatory neurons with the central nervous system (31). Study has demonstrated that hypermethylation of its promoter region is associated with reduced expression in non-neural tissues, such as lymphocytes (32). Its role in cancer appears complex: depletion in KRAS-mutant cancers triggers hyperactivation of the MAPK/ERK pathway and subsequent cell death (33), whereas the lncRNA SHANK3 has been linked to poor prognosis in gastric cancer (14). Our detection of SHANK3 hypermethylation in EC may indicate a novel tumor-suppressive function or cancer-specific pathway disruption, which warrants further investigation.

FHL1, located on the X chromosome, is a key tumor suppressor gene that regulates cell adhesion, proliferation, and differentiation (34). In various cancers, including breast cancer and lung cancer, FHL1 expression is significantly downregulated. Promoter hypermethylation leads to gene silencing, thereby causing the loss of its tumor-suppressing function (35–37). In gastrointestinal cancers, FHL1 methylation forms part of an “epigenetic field defect” associated with risk factors like Helicobacter pylori infection (38). The hypermethylation of FHL1 observed in EC is consistent with its well-established role as a tumor suppressor in other cancer types. This study employed cervical scraping as a diagnostic tool, thereby avoiding the limitations of invasive procedures. In clinical practice, re-testing for methylation in patients with abnormal ultrasound findings can reduce the need for multiple unnecessary biopsies. In the future, the extent of methylation marker aberrations might be correlated with pathological staging to guide the selection of EC treatment regimens.

### Limitations

While this study provides promising results, several limitations should be acknowledged. First, the sample size is small (20 EC patients and 20 controls), likely due to limited availability of eligible samples and clinical data, which restricts statistical power and generalizability; future studies should include larger, multicenter cohorts. Second, women with endometrial lesions or reproductive disorders were excluded, probably to reduce clinical heterogeneity, limiting applicability to broader populations; future research should incorporate diverse patient groups. Third, MCTA-Seq is technically complex and costly, reflecting high requirements for library preparation, sequencing, and bioinformatic analysis, which may hinder routine clinical use; future work should simplify the workflow and improve cost-effectiveness. Fourth, no functional experiments were performed to validate the biological roles of BAHCC1, SHANK3, and FHL1, as the study focused on biomarker discovery, limiting mechanistic interpretation; future studies should explore these markers *in vitro* and *in vivo*. Finally, only internal validation was conducted, likely due to limited access to additional well-characterized cohorts; future research should include independent external validation.

## Conclusions

This study explored a sequential combination strategy of TVUS and DNA methylation detection in cervical scrapings, constructing a non-invasive approach for EC screening. This strategy has improved the diagnostic performance (AUC = 0.950) and appears to be clinically feasible, suggesting a potential option for early EC detection and intervention. Functional analysis of DMGs provided preliminary epigenetic insights into EC pathogenesis, and the three core methylation biomarkers identified may serve as potential molecular targets for non-invasive diagnosis.

However, this is a single-center exploratory study with a small sample size. The results need to be validated in large-scale, multi-center independent cohorts. In addition, the clinical promotion of MCTA-Seq technology needs further optimization, and the biological functions and regulatory mechanisms of the three core biomarkers in EC need in-depth research. Future research will expand the sample size to conduct multi-center prospective studies and carry out functional experiments of the biomarkers to provide more sufficient evidence for the clinical application and mechanism research of this strategy.

## Data Availability

All datasets of this study have been uploaded to the public database The Genome Sequence Archive for Human (GSA-Human) with the accession number HRA010662, which can be freely accessed through the public database, and the data can also be obtained from the corresponding authors upon reasonable request.
