# Supplementary material for: Application of ultrasound and methylation omics detection in the diagnosis of endometrial cancer
Source: Front Oncol. 2026 Jun 3;16:1803441. doi: 10.3389/fonc.2026.1803441 (PMC13272088; doi:10.3389/fonc.2026.1803441)
Supplement: Supplementary file 1 [file Table1.docx]

Supplementary material

| Differentially methylated CpG sites |
| --- |
| EGR2-862_8_8 |
| EGR2-862_8_9 |
| SHISA2-4062_2_9 |
| GDI1-26764_1_9 |
| GDI1-26764_1_10 |
| PPAP2C-10294_3_5 |
| SHISA2-4062_2_10 |
| HOXD9-16791_2_4 |
| --22104_1_2 |
| EGR2-862_8_10 |
| SOX1-3812_3_5 |
| SOX1-3812_3_4 |
| SHISA2-4062_2_8 |
| FZD2-7955_2_1 |
| GDI1-26764_1_7 |
| CRTAC1-1135_3_6 |
| C1orf61-12547_1_4 |
| FZD2-7955_2_2 |
| ZPBP2-7805_1_8 |
| ZPBP2-7805_1_9 |
| --3058_1_3 |
| MACROD1-1969_2_1 |
| SFMBT2-944_7_6 |
| --158_1_1 |
| SOX1-3812_3_6 |
| NRN1-22486_1_7 |
| --15131_1_7 |
| --15131_1_5 |
| JSRP1-10195_1_6 |
| --15131_1_6 |
| JSRP1-10195_1_7 |
| --4184_2_1 |
| --4184_2_2 |
| GDI1-26764_1_8 |
| FZD2-7955_2_3 |
| --6474_2_9 |
| --23119_2_1 |
| OPCML-1419_1_1 |
| CELSR3-18847_2_5 |
| MACROD1-1969_2_2 |
| DBX1-1553_1_3 |
| GDI1-26764_1_6 |
| GPM6B-26654_3_8 |
| --9418_5_2 |
| --9418_5_1 |
| --158_1_2 |
| --20989_2_8 |
| --4971_1_3 |
| FBXL21-20376_2_8 |
| KLHDC7B-16275_2_5 |
| PLLP-6693_1_1 |
| SHISA2-4062_2_6 |
| --3221_1_4 |
| ZNF503-AS2-977_2_2 |
| MTSS1-24353_2_4 |
| OPCML-1419_1_2 |
| SHISA2-4062_2_7 |
| --9194_1_10 |
| EGR4-17760_1_1 |
| EGR4-17760_1_2 |
| --9418_5_3 |
| MBTPS2-26848_1_6 |
| MBTPS2-26848_1_5 |
| CNTFR-26032_2_1 |
| BRCC3-26785_2_10 |
| --9418_5_4 |
| SLC6A11-18007_2_5 |
| ECEL1-17149_2_8 |
| CNTFR-26032_2_2 |
| GABBR2-25234_8_3 |
| OPCML-1419_1_3 |
| CAMTA1-14229_5_3 |
| CALN1-23935_3_3 |
| BAI1-24472_2_3 |
| --22950_1_3 |
| CAMTA1-14229_5_4 |
| --4971_1_4 |
| OTUD7A-5252_3_8 |
| GRB10-23674_1_8 |
| ADAM32-24869_1_5 |
| BAI1-24472_2_5 |
| --20989_2_7 |
| ZYG11A-14050_1_9 |
| GRB10-23674_1_9 |
| GABRA4-19802_2_4 |
| --4971_1_5 |
| GRB10-23674_1_10 |
| SH3KBP1-26838_4_8 |
| SOX1-3812_3_7 |
| CNTFR-26032_2_3 |
| LINC00605-4401_1_5 |
| MIR124-3-15066_5_8 |
| NKX2-2-14482_7_7 |
| SYN2-18063_4_1 |
| CTNND2-20225_4_9 |
| CNTFR-26032_2_4 |
| FHL1-26624_1_4 |
| PCDHA1-20451_1_6 |
| MIR124-3-15066_5_9 |
| ADAM32-24869_1_8 |
| T-21795_1_4 |
| PTH1R-18815_1_3 |
| MIR4683-665_1_5 |
| CAMTA1-14229_5_2 |
| MIR4683-665_1_6 |
| KBTBD11-24705_8_9 |
| --22950_2_2 |
| JSRP1-10195_1_8 |
| SLCO6A1-20163_1_9 |
| CNTN4-18651_3_6 |
| --23119_2_2 |
| MAF1-24592_1_10 |
| PDE10A-21786_2_3 |
| --17373_1_8 |
| INSM1-14470_6_1 |
| POU3F1-13831_3_3 |
| POU3F1-13831_3_2 |
| --13327_1_5 |
| ECEL1-17149_2_9 |
| HMX2-280_2_2 |
| --9418_5_5 |
| LINC00605-4401_1_6 |
| MYO10-20711_2_2 |
| LINC00605-4401_1_7 |
| SFMBT2-944_7_7 |
| SOX13-12977_1_2 |
| SOX13-12977_1_1 |
| LINC00605-4401_1_8 |
| --17373_1_7 |
| SOX1-3812_3_8 |
| --22104_1_4 |
| --22104_1_3 |
| SORCS1-154_1_6 |
| --695_1_3 |
| --695_1_4 |
| OPCML-1419_1_6 |
| FAM19A5-16160_2_6 |
| --14264_1_2 |
| JSRP1-10195_1_9 |
| CELSR3-18847_2_6 |
| CELSR3-18847_2_7 |
| OPCML-1419_1_4 |
| PIM2-27048_1_5 |
| POU6F2-23549_1_1 |
| CNTFR-26032_2_5 |
| RAI2-26825_1_9 |
| GPR26-283_2_5 |
| VIPR2-23267_1_9 |
| SCRT1-24605_9_2 |
| ZFYVE28-19650_2_9 |
| NKX2-2-14482_1_6 |
| CLYBL-3727_1_4 |
| GDI1-26764_1_5 |
| --14264_1_3 |
| TNFSF11-4169_1_4 |
| OBSCN-13274_3_3 |
| CAMTA1-14229_5_1 |
| --19064_1_7 |
| ECEL1-17149_2_2 |
| ROBO3-1344_2_4 |
| FAM110B-24976_2_3 |
| LINC00605-4401_1_9 |
| CLYBL-3727_2_3 |
| CCDC39-18467_1_2 |
| RANBP9-21600_3_3 |
| --19564_1_1 |
| LOC101055625-3132_1_9 |
| LOC101055625-3132_1_8 |
| --17725_1_1 |
| KBTBD11-24705_2_2 |
| DCAF12L1-26559_1_8 |
| GALNT14-17452_1_5 |
| FEV-17030_3_1 |
| KLHL1-4250_1_6 |
| T-21795_1_5 |
| MYO10-20711_2_4 |
| MYO10-20711_2_3 |
| FAM110B-24976_2_4 |
| RASAL2-12787_1_1 |
| CECR6-15598_2_6 |
| TERT-20305_3_1 |
| --27059_2_8 |
| ZIM2-11643_1_5 |
| ERICH1-25001_1_8 |
| LOC646862-11730_1_2 |
| CCDC39-18467_1_3 |
| MTSS1-24353_2_5 |
| SOX1-3812_3_9 |
| --26957_2_4 |
| --15131_1_4 |
| UNCX-22826_3_4 |
| NPBWR1-24940_2_4 |
| CAMTA1-14229_4_1 |
| CAMTA1-14229_4_2 |
| ELOVL4-22561_2_1 |
| --17724_1_1 |
| BAI1-24472_2_6 |
| --14327_3_7 |
| SOX18-15172_1_7 |
| C17orf51-7540_1_1 |
| SLC10A3-26773_1_7 |
| ELOVL4-22561_2_2 |
| NPBWR1-24940_2_3 |
| FAM110B-24976_2_5 |
| GALNT14-17452_1_6 |
| --324_3_3 |
| SCGB3A1-20930_2_1 |
| LINGO3-10207_3_10 |
| --26885_1_10 |
| DBX1-1553_1_2 |
| TEX28-26749_1_4 |
| TEX28-26749_1_3 |
| TEX28-26749_1_2 |
| ELOVL4-22561_2_3 |
| TNFSF11-4169_1_2 |
| FOXA1-4712_2_2 |
| ZNF512B-15165_2_2 |
| ABCA1-25270_1_6 |
| SLC10A3-26773_1_8 |
| --4184_1_7 |
| ARHGEF10-24686_1_3 |
| --18669_1_2 |
| --22251_2_9 |
| POU3F1-13831_3_1 |
| BAHCC1-8721_2_1 |
| ECEL1-17149_2_10 |
| LOC728613-20692_1_1 |
| LOC728613-20692_1_2 |
| C17orf51-7540_1_2 |
| CHST3-931_1_1 |
| --158_1_3 |
| SORCS1-154_1_8 |
| SORCS1-154_1_9 |
| --4403_2_7 |
| --16477_8_1 |
| CHST3-931_1_3 |
| CHST3-931_1_2 |
| FHL1-26624_1_3 |
| SORCS1-154_1_7 |
| CD99L2-26685_4_5 |
| CHST3-931_1_4 |
| LPL-24710_2_8 |
| TRIM54-17417_3_4 |
| LINC00605-4401_1_10 |
| --1718_2_3 |
| CHST3-931_1_5 |
| PRSS41-6258_4_6 |
| SOX18-15172_1_6 |
| TFAP2E-13770_2_1 |
| --25865_2_7 |
| SHISA6-7341_7_5 |
| RASIP1-11185_3_9 |
| CTNND2-20225_4_8 |
| C17orf51-7540_1_3 |
| SFMBT2-944_7_5 |
| BAI1-24472_2_4 |
| --695_1_1 |
| --695_1_2 |
| TBX4-8273_1_5 |
| --26928_3_9 |
| TBX4-8273_1_6 |
| --5674_6_1 |
| MATN4-14774_1_9 |
| ATP6V1C2-16352_3_3 |
| ATP6V1C2-16352_3_2 |
| STAC-18706_1_4 |
| HOXC12-3391_1_2 |
| SCGB3A1-20930_2_2 |
| FLJ42709-21351_4_8 |
| SALL3-9342_3_10 |
| --19064_1_8 |
| SBNO2-9551_1_9 |
| --14327_3_2 |
| STAC-18706_2_6 |
| PPAP2C-10294_3_6 |
| CLYBL-3727_2_4 |
| GPM6B-26654_3_9 |
| --506_1_9 |
| --506_1_10 |
| MINK1-8136_3_1 |
| --3221_2_3 |
| --25865_2_4 |
| ZFYVE28-19650_2_8 |
| NKX2-2-14482_8_8 |
| LPL-24710_2_10 |
| LPL-24710_2_9 |
| SOX13-12977_1_3 |
| --6902_2_4 |
| HMX2-280_2_3 |
| LINGO3-10207_1_5 |
| ZNF470-11632_1_6 |
| ZNF470-11632_1_5 |
| SOX18-15172_1_5 |
| --13327_1_4 |
| --4927_1_7 |
| --4927_1_6 |
| GALNT14-17452_1_7 |
| GALNT14-17452_1_8 |
| --23119_3_6 |
| ZYG11A-14050_1_10 |
| ST8SIA3-9198_1_10 |
| ADRB3-24853_1_3 |
| PPAP2C-10294_3_7 |
| --19330_2_6 |
| TONSL-24620_1_6 |
| HHIPL1-4318_1_2 |
| HHIPL1-4318_1_3 |
| HHIPL1-4318_1_1 |
| WDR45-27056_2_3 |
| HCFC1-26744_1_3 |
| ATP9B-9345_1_7 |
| UTF1-474_1_5 |
| --25865_2_5 |
| LOC728613-20692_1_3 |
| CAMTA1-14229_2_1 |
| --26960_3_3 |
| POU3F2-22623_2_3 |
| ECEL1-17149_2_6 |
| ECEL1-17149_2_4 |
| ECEL1-17149_2_5 |
| ECEL1-17149_2_3 |
| --4971_1_1 |
| CDH22-14814_2_1 |
| PHOX2A-2281_1_9 |
| LRIG3-3516_1_2 |
| ARHGEF10-24686_1_4 |
| LMF1-5883_4_5 |
| TNFSF11-4169_1_1 |
| TFAP2A-21416_1_1 |
| RORB-26247_2_3 |
| RNF39-22094_1_2 |
| RNF39-22094_1_1 |
| --8272_1_10 |
| EGR4-17760_1_3 |
| --6474_2_8 |
| HEYL-13846_1_10 |
| HEYL-13846_1_9 |
| --2413_1_6 |
| --2413_1_4 |
| --2413_1_5 |
| POU3F2-22623_2_1 |
| BMP7-14904_1_5 |
| --16477_8_3 |
| --16477_8_2 |
| CNTFR-26032_2_6 |
| ADRB3-24853_1_4 |
| CAPN2-13183_3_4 |
| CAPN2-13183_3_3 |
| CAPN2-13183_3_2 |
| --17725_1_2 |
| NKX2-2-14482_7_6 |
| --17373_1_9 |
| SLC38A3-18893_1_2 |
| SLC38A3-18893_1_1 |
| CLYBL-3727_1_2 |
| VPS16-14542_1_8 |
| LOC101055625-3132_1_10 |
| CRTAC1-1135_3_5 |
| ZNF470-11632_1_7 |
| POU3F2-22623_2_2 |
| TNFSF11-4169_1_3 |
| --11113_2_4 |
| MBTPS2-26848_1_4 |
| --9281_3_1 |
| CLYBL-3727_2_2 |
| --9238_1_6 |
| ADRA1A-24794_3_1 |
| PLXNC1-3689_2_3 |
| LRIG3-3516_1_3 |
| ZNF71-11633_1_1 |
| POU3F2-22623_2_4 |
| INSM1-14470_6_2 |
| PPAP2C-10294_3_4 |
| PDE10A-21786_2_1 |
| --7241_4_1 |
| NTRK3-5758_1_4 |
| NKX2-2-14482_1_7 |
| LMF1-5883_4_6 |
| RPS6KA4-2001_1_9 |
| OBSCN-13274_3_1 |
| ZNF71-11633_1_2 |
| --4927_1_5 |
| NTHL1-6119_1_5 |
| NKX2-2-14482_7_4 |
| --24466_3_2 |
| ZNF503-AS2-977_17_9 |
| ASIC2-7677_2_4 |
| RANBP9-21600_3_1 |
| --14264_1_4 |
| TUSC3-24668_1_5 |
| TUSC3-24668_1_4 |
| PDE4B-14177_1_4 |
| PDE4B-14177_1_3 |
| RNF180-21177_1_2 |
| ADRA1A-24794_3_2 |
| --78_4_3 |
| ONECUT3-9949_2_4 |
| TUSC3-24668_1_6 |
| --19694_1_3 |
| --3637_2_2 |
| GALNT14-17452_1_9 |
| BAHCC1-8721_7_2 |
| ASIC2-7677_2_5 |
| --22950_1_4 |
| --25097_1_2 |
| LRRN1-18723_3_1 |
| --25097_1_1 |
| UNCX-22826_3_5 |
| RANBP9-21600_3_2 |
| CAMTA1-14229_5_5 |
| MCHR2-21389_2_4 |
| DRD4-1965_3_10 |
| NTRK3-5758_1_5 |
| ARHGEF10-24686_1_2 |
| LRIT1-1024_1_6 |
| KLHDC7B-16275_2_4 |
| --22104_1_5 |
| IRS2-3760_7_4 |
| NKX2-2-14482_1_5 |
| LRRN1-18723_3_2 |
| RGPD8-16396_1_9 |
| --19330_4_5 |
| BEGAIN-4342_2_5 |
| MMP25-6385_4_1 |
| SLCO6A1-20163_1_6 |
| ALG13-26486_2_5 |
| CES4A-6769_1_3 |
| --15983_3_2 |
| --9418_2_9 |
| AMN-4390_4_1 |
| LINC00461-21334_2_3 |
| LINC00461-21334_2_2 |
| PPARG-18087_1_3 |
| FAM19A5-16163_2_8 |
| COL23A1-20874_1_1 |
| ALG13-26486_2_6 |
| COL23A1-20874_1_2 |
| C1QL2-16453_3_9 |
| SLC6A11-18007_3_7 |
| --20755_1_4 |
| REREP3-5154_3_4 |
| ALG13-26486_2_3 |
| CD99L2-26685_3_8 |
| --13327_1_10 |
| CAPN2-13183_3_5 |
| --2507_3_4 |
| ECEL1-17149_2_7 |
| --13327_1_6 |
| MTSS1-24353_2_3 |
| --14552_2_1 |
| EPHA7-22613_1_2 |
| GABRG3-5195_1_6 |
| NTM-1417_5_6 |
| LMF1-5883_4_7 |
| LINGO3-10207_1_4 |
| --17094_1_2 |
| CHST3-931_2_1 |
| FOXA1-4712_2_1 |
| LATS2-4008_2_9 |
| --24927_2_1 |
| --24927_2_2 |
| LINC00605-4401_1_4 |
| TTC40-425_1_10 |
| DRD4-1965_4_9 |
| DRD4-1965_4_10 |
| --23126_3_2 |
| HEYL-13846_1_6 |
| CHST3-931_2_2 |
| ITGA11-5567_1_8 |
| ITGA11-5567_1_7 |
| ITGA11-5567_1_6 |
| ITGA11-5567_1_5 |
| MTSS1-24353_2_2 |
| MINK1-8136_3_2 |
| PSEN2-13229_1_2 |
| --15983_3_3 |
| --324_3_1 |
| ARMCX4-26426_1_8 |
| BTNL9-20943_2_1 |
| --924_2_7 |
| EGR2-862_8_7 |
| LOC254559-5777_1_7 |
| BTNL9-20943_2_2 |
| OPLAH-24584_3_7 |
| RNF39-22094_1_3 |
| ALDH1A3-5116_3_10 |
| CPPED1-5963_1_3 |
| CPPED1-5963_1_2 |
| --19869_3_6 |
| UNCX-22826_11_5 |
| CKAP4-2551_2_1 |
| ONECUT3-9949_2_2 |
| RLTPR-6803_2_6 |
| SOX1-3812_3_10 |
| ALG13-26486_2_4 |
| HEYL-13846_1_8 |
| HEYL-13846_1_7 |
| MOB2-1506_2_3 |
| ADRB1-185_1_5 |
| RNF220-13943_1_7 |
| --695_1_5 |
| --695_1_6 |
| EGR4-17760_1_4 |
| FOXB1-5478_2_1 |
| --23126_3_3 |
| CNTFR-26032_1_2 |
| ARHGEF10-24686_1_1 |
| CHST3-931_2_3 |
| --22950_2_4 |
| --22950_2_3 |
| --2507_3_5 |
| FLJ37453-12641_1_7 |
| --8272_1_5 |
| GDNF-21048_1_1 |
| --25097_1_3 |
| C9orf172-25809_7_6 |
| TXNRD1-2534_1_3 |
| ZSCAN1-11704_2_2 |
| GPR26-283_2_4 |
| RAI1-7444_1_4 |
| MINK1-8136_4_1 |
| NKX2-2-14482_8_1 |
| CLYBL-3727_2_5 |
| OBSCN-13274_3_2 |
| SVEP1-25298_1_7 |
| MOB2-1506_2_4 |
| FAM19A5-16163_3_10 |
| CLYBL-3727_1_3 |
| --10404_1_4 |
| PTPRM-9308_1_5 |
| --19064_1_6 |
| LRIG3-3516_1_1 |
| PPAP2C-10294_3_8 |
| --2648_1_8 |
| RAI1-7444_1_3 |
| --16477_7_3 |
| ARHGEF10-24686_1_5 |
| ZNF529-10551_5_1 |
| ZNF529-10551_5_3 |
| ZNF529-10551_5_2 |
| JSRP1-10195_1_5 |
| --23126_3_4 |
| OPCML-1419_1_5 |
| ARHGAP27-7988_2_7 |
| MAF1-24592_1_8 |
| COL9A3-15038_4_5 |
| BTNL9-20943_2_3 |
| EPCAM-17578_3_1 |
| TERT-20305_3_2 |
| --19694_1_4 |
| ADRB3-24853_1_5 |
| PPAP2C-10294_6_1 |
| SERP2-4179_4_9 |
| GPR26-283_2_6 |
| OPLAH-24584_1_6 |
| FAM43A-18584_2_6 |
| JSRP1-10195_1_4 |
| PDX1-4078_1_1 |
| --19784_2_10 |
| RPL39-26527_1_8 |
| --695_1_8 |
| --20096_3_7 |
| TDH-24281_2_3 |
| OPLAH-24584_3_4 |
| SLC22A18-1616_1_1 |
| FAM65A-6798_3_1 |
| ONECUT1-5437_2_3 |
| PHF17-19285_1_2 |
| PHF17-19285_1_1 |
| NPBWR1-24940_3_5 |
| SERP2-4179_4_10 |
| ADAMTS2-20887_2_4 |
| DRAXIN-12177_1_1 |
| FOXC2-7061_2_10 |
| FOXC2-7061_2_9 |
| FOXC2-7061_2_8 |
| PTPRN2-23243_4_4 |
| GPRASP1-26435_1_9 |
| GPRASP1-26435_1_8 |
| EXOC3-21093_1_8 |
| SLIT2-19634_1_1 |
| TDRD12-10369_1_9 |
| TDRD12-10369_1_8 |
| DNAJC6-14172_2_6 |
| DNAJC6-14172_2_7 |
| TDRD12-10369_1_7 |
| DNAJC6-14172_2_5 |
| TDRD12-10369_1_6 |
| LGR4-1597_1_3 |
| LGR4-1597_1_2 |
| LGR4-1597_1_1 |
| FOXA1-4712_2_3 |
| --26365_5_3 |
| --25865_2_2 |
| --23119_2_3 |
| MCC-20220_3_10 |
| MCC-20220_3_7 |
| MCC-20220_3_9 |
| MCC-20220_3_8 |
| ADAM32-24869_1_1 |
| CNTFR-26032_1_1 |
| --3637_1_1 |
| ELOVL4-22561_2_4 |
| PHOX2A-2281_1_8 |
| --27059_2_7 |
| --27059_2_6 |
| NAE1-6759_1_5 |
| TPBGL-2325_2_3 |
| TPBGL-2325_2_2 |
| TPBGL-2325_2_1 |
| ARHGAP27-7988_2_6 |
| FOXE1-25224_2_10 |
| LOC284801-14531_1_9 |
| KLHL1-4250_1_3 |
| ATP8B3-9979_1_3 |
| --3221_3_6 |
| FGF13-26648_2_8 |
| GSTM3-12075_1_4 |
| GSTM3-12075_1_3 |
| GSTM3-12075_1_2 |
| FBXO27-10669_1_1 |
| GSTM3-12075_1_1 |
| SNX22-5506_1_3 |
| SNX22-5506_1_2 |
| CRTAC1-1135_4_3 |
| PTGFR-14244_1_1 |
| PDE10A-21786_2_4 |
| AMN-4389_2_4 |
| OTUD7A-5252_3_9 |
| FAM19A5-16160_2_8 |
| FAM19A5-16160_2_9 |
| ZNF470-11632_1_2 |
| ZSCAN1-11704_2_4 |
| DNAJC6-14172_2_8 |
| MOB2-1506_2_5 |
| BTNL9-20943_2_4 |
| TRIM54-17417_3_7 |
| NTHL1-6119_1_6 |
| CHST3-931_2_4 |
| MEGF6-13750_1_1 |
| ROBO3-1344_2_5 |
| NTSR2-16430_1_1 |
| GABRA4-19802_2_5 |
| KLHL1-4250_1_2 |
| --24649_1_8 |
| EBF3-342_5_7 |
| PHF6-26599_1_6 |
| TRIM54-17417_3_5 |
| TRIM54-17417_3_6 |
| KLHL1-4250_1_5 |
| RNF180-21177_1_1 |
| --15131_3_4 |
| --14483_2_6 |
| MPST-15900_3_10 |
| ADAM32-24869_1_6 |
| TRPS1-24302_1_2 |
| LINC00629-26603_1_8 |
| --158_1_4 |
| --14264_1_1 |
| HCFC1-26744_1_4 |
| HCFC1-26744_1_5 |
| PDE4C-10028_1_1 |
| FAM19A5-16163_2_9 |
| --9281_2_1 |
| MYO3A-602_1_8 |
| MYO3A-602_1_9 |
| MYO3A-602_1_10 |
| PTPRN2-23243_4_5 |
| TCTEX1D1-14185_1_9 |
| --23452_1_4 |
| LINC00667-9188_1_6 |
| STARD8-27170_1_5 |
| PTH1R-18815_1_4 |
| ZYG11A-14050_3_1 |
| UNCX-22826_5_7 |
| ADAM32-24869_1_4 |
| --25097_2_3 |
| ZSCAN1-11704_2_1 |
| --22950_1_1 |
| --19782_1_4 |
| AFG3L1P-7299_2_4 |
| AFG3L1P-7299_2_3 |
| --2507_3_6 |
| COL23A1-20874_1_3 |
| --324_3_2 |
| RIMS2-24239_1_2 |
| MROH1-24595_1_7 |
| CAMTA1-14229_3_7 |
| CAMTA1-14229_3_8 |
| CAMTA1-14229_3_9 |
| CDO1-20233_1_8 |
| --4216_2_4 |
| FOXE1-25223_1_1 |
| --17141_2_10 |
| GDNF-21048_1_2 |
| --2507_3_7 |
| TCTEX1D1-14185_1_8 |
| --20985_1_7 |
| EBF2-24785_1_2 |
| TEX26-AS1-4114_2_8 |
| --9194_1_9 |
| --22327_1_1 |
| --701_1_3 |
| SBK1-6252_1_9 |
| --6940_1_9 |
| CECR6-15598_2_8 |
| CECR6-15598_2_7 |
| CNTFR-26032_2_7 |
| ZNF71-11633_1_3 |
| --14327_3_1 |
| CASK-26988_1_2 |
| CASK-26988_1_1 |
| --7218_1_1 |
| EBF3-342_11_1 |
| KLHL31-22463_1_2 |
| --4403_2_8 |
| MYO10-20711_2_1 |
| SCRT2-15193_1_2 |
| --21796_3_3 |
| --21796_3_2 |
| RNF220-13943_1_8 |
| PTPRU-13624_3_2 |
| BEGAIN-4342_2_3 |
| --14264_1_5 |
| FOXP2-22775_4_1 |
| FBXO43-24208_1_1 |
| CNTFR-26032_1_3 |
| --695_1_10 |
| --695_1_9 |
| PLEKHG4-6790_4_1 |
| EBF3-342_11_2 |
| PRSS56-17151_2_3 |
| LINC00605-4401_1_3 |
| POU3F2-22623_2_5 |
| GABRG3-5195_1_7 |
| --13327_1_7 |
| DCAF12L1-26559_1_9 |
| LINC00667-9188_1_8 |
| --1326_1_6 |
| --22327_1_2 |
| EBF2-24785_1_1 |
| FOXA1-4712_2_4 |
| ADRB1-185_1_4 |
| ZNF470-11632_1_8 |
| EBF3-342_11_3 |
| --20761_2_4 |
| KLHL1-4250_1_1 |
| PCDHGA1-20521_1_2 |
| FAM110C-17562_2_1 |
| BEGAIN-4342_2_4 |
| FOXP2-22775_4_3 |
| C12orf56-3540_1_8 |
| LOC100190940-2884_2_1 |
| ZNF470-11632_1_4 |
| RHOU-13286_26_2 |
| IGSF9B-1430_1_5 |
| PRDM16-13628_11_8 |
| EBF3-342_11_5 |
| EBF3-342_11_4 |
| --21238_1_1 |
| UNCX-22826_3_3 |
| RNF207-14133_3_10 |
| --16322_1_3 |
| DRD4-1965_4_6 |
| --277_1_2 |
| POU3F2-22623_4_6 |
| LOX-20260_1_2 |
| --3637_2_3 |
| --26651_1_10 |
| --16308_2_4 |
| --16308_2_3 |
| --16308_2_2 |
| KLHL31-22463_2_3 |
| NKX2-2-14482_7_5 |
| LOC286177-24973_1_5 |
| --15316_2_9 |
| --15316_2_8 |
| --15316_2_6 |
| --15316_2_7 |
| --14330_2_9 |
| --14330_2_8 |
| HHIPL1-4318_1_4 |
| --16102_1_3 |
| COL5A1-25679_1_7 |
| YDJC-15718_1_5 |
| FAM19A5-16160_2_7 |
| GABRA4-19802_2_1 |
| AGAP1-17180_1_5 |
| --4440_1_6 |
| --3058_2_4 |
| TMEM132E-7681_1_6 |
| --15189_2_10 |
| CPSF3-17915_1_5 |
| DRD4-1965_4_8 |
| DRD4-1965_4_7 |
| --19869_3_7 |
| --19869_3_8 |
| --19869_3_9 |
| --19869_3_10 |
| ZNF71-11633_1_5 |
| ZNF71-11633_1_4 |
| --26518_3_9 |
| ICAM5-9491_3_4 |
| ICAM5-9491_3_5 |
| PAPLN-4918_1_2 |
| PDE10A-21786_2_2 |
| FAM53A-19484_1_5 |
| FAM53A-19484_1_6 |
| IMPDH1-22845_1_1 |
| --16308_2_5 |
| ADAMTS2-20887_2_8 |
| ADAMTS2-20887_2_7 |
| EBF3-342_11_6 |
| GDI1-26764_1_4 |
| --25442_2_1 |
| --23119_2_4 |
| SEMA5B-18080_1_9 |
| MINK1-8136_4_2 |
| VPS16-14542_1_10 |
| VPS16-14542_1_9 |
| OPLAH-24584_3_5 |
| ADRB3-24853_1_6 |
| HOXB3-8064_2_6 |
| LINGO3-10207_1_8 |
| FAM163A-12801_4_6 |
| NIPAL4-20654_1_7 |
| NIPAL4-20654_1_6 |
| ZNF503-AS2-977_16_5 |
| BMP4-4774_5_5 |
| NPY5R-19469_4_8 |
| BNC1-5732_1_3 |
| HSPA12A-204_1_1 |
| VPS16-14542_2_2 |
| --4772_1_6 |
| POU6F2-23549_1_3 |
| SP9-16762_1_2 |
| HMGB3-26686_2_6 |
| AMN-4390_4_2 |
| --14330_1_7 |
| IRS2-3760_7_5 |
| --9140_1_5 |
| PCDHA1-20453_1_2 |
| EXOC3L4-4397_1_10 |
| --18669_1_3 |
| --26960_3_4 |
| MIR4683-665_1_7 |
| SOX1-3813_1_6 |
| C2orf40-16340_2_4 |
| C2orf40-16340_2_3 |
| C2orf40-16340_2_2 |
| C2orf40-16340_2_1 |
| ZNF503-AS2-977_16_4 |
| COL23A1-20874_1_4 |
| TERT-20305_3_3 |
| PTPRN2-23195_18_8 |
| --3221_1_5 |
| PARP6-5592_2_1 |
| PARP6-5592_2_2 |
| RGS10-243_2_6 |
| PRSS36-6403_2_1 |
| SLCO6A1-20163_1_7 |
| UNCX-22826_10_2 |
| RIMS2-24239_1_3 |
| PTPRU-13624_3_1 |
| CHRNA3-5688_5_3 |
| CHRNA3-5688_5_2 |
| CHRNA3-5688_5_1 |
| TRIM54-17417_3_8 |
| PTPRN2-23195_3_8 |
| EBF3-342_11_7 |
| ECEL1-17149_2_1 |
| BHLHE23-15053_3_3 |
| LHX5-2646_1_2 |
| --10605_1_3 |
| OTUD7A-5252_3_10 |
| GAD2-604_1_2 |
| INPP5A-401_1_8 |
| ADAM32-24869_1_3 |
| CSDA-2572_1_1 |
| CHST3-931_1_6 |
| NKX2-2-14482_8_2 |
| LINC00577-21425_1_3 |
| SHROOM2-27268_3_5 |
| PTER-549_1_4 |
| PACS1-2102_2_1 |
| CBX4-8620_3_3 |
| PTPRN2-23195_18_7 |
| LMF1-5883_4_4 |
| --23058_1_8 |
| JPH3-7090_13_4 |
| SLCO6A1-20163_1_8 |
| RAI1-7444_1_2 |
| --13481_1_8 |
| --26651_2_7 |
| RPS6KA4-2001_1_10 |
| ANKRD24-10823_1_10 |
| ONECUT3-9949_2_5 |
| BAHCC1-8721_7_3 |
| EVC-19873_1_4 |
| EVC-19873_1_3 |
| PLCD1-18717_3_5 |
| ITGA11-5567_1_4 |
| HEYL-13846_1_5 |
| --18669_1_1 |
| PTER-549_1_3 |
| --924_2_8 |
| --924_2_9 |
| --23126_3_5 |
| GBX2-17186_3_6 |
| CNTFR-26032_1_4 |
| PDE1C-23503_3_6 |
| DNAJC6-14172_2_9 |
| MOB2-1481_1_3 |
| FGF13-26648_2_9 |
| POU3F2-22623_2_7 |
| RNF220-13943_1_9 |
| PAX9-4707_1_2 |
| MN1-15800_1_7 |
| STARD8-27170_1_2 |
| --26651_2_4 |
| POU6F2-23549_1_2 |
| CD8A-17845_1_6 |
| --26960_3_5 |
| FGF13-26645_3_9 |
| ZPBP2-7805_1_7 |
| TBXA2R-10466_1_7 |
| KCNS2-25207_3_9 |
| FOXP2-22775_4_2 |
| NKX2-2-14482_7_2 |
| LINC00466-14145_3_3 |
| IRS2-3760_7_6 |
| SCRT1-24605_10_6 |
| --19900_1_9 |
| SLC6A11-18007_3_10 |
| --15777_2_5 |
| CSDA-2572_1_2 |
| PTPRN2-23195_3_9 |
| --25734_4_2 |
| ST8SIA3-9198_1_9 |
| --8272_2_6 |
| PDE9A-15383_1_9 |
| FEZF2-19016_1_5 |
| KCND2-22795_1_4 |
| EPHA7-22613_1_1 |
| ZYG11A-14050_1_8 |
| RPS6KA3-26842_5_8 |
| --3221_3_7 |
| ZBTB10-25102_1_1 |
| UNCX-22826_3_6 |
| --27187_2_7 |
| HHEX-1076_1_1 |
| LOC100288524-23509_2_7 |
| --22950_1_2 |
| PACS1-2102_4_8 |
| PACS1-2102_4_7 |
| REREP3-5154_4_4 |
| FAM110C-17562_2_2 |
| UNCX-22826_5_4 |
| PRR23C-18276_1_10 |
| SLC39A4-24614_1_5 |
| --22876_1_6 |
| MAFB-14729_1_1 |
| AFG3L1P-7299_2_6 |
| AFG3L1P-7299_2_5 |
| CES1P1-6661_1_3 |
| POU3F2-22623_2_6 |
| PCDHGA1-20521_1_3 |
| NTM-1417_3_8 |
| DGKQ-20144_3_1 |
| --19784_4_10 |
| DOK6-9253_3_4 |
| CNTFR-26032_1_5 |
| --22950_1_5 |
| --16322_1_2 |
| BARHL1-25611_1_1 |
| PRDM5-19247_1_2 |
| BARHL2-14324_1_4 |
| --14483_2_5 |
| GAL3ST3-2099_2_10 |
| GAL3ST3-2099_2_9 |
| --4772_1_4 |
| BARHL2-14324_1_3 |
| LRIT1-1024_1_7 |
| NKX2-2-14482_7_3 |
| OPLAH-24584_3_6 |
| ZNF503-AS2-977_2_1 |
| LINGO3-10207_3_9 |
| FAM84B-24360_1_4 |
| --14330_1_3 |
| TNFSF11-4169_1_5 |
| OTUD7A-5252_4_7 |
| --4216_2_3 |
| LOC100131655-9292_1_1 |
| STARD8-27170_1_3 |
| GPR26-283_2_2 |
| TPBG-22568_1_5 |
| PRSS41-6258_1_9 |
| CCDC39-18467_1_1 |
| FAM53A-19484_1_4 |
| MIR3687-15570_1_7 |
| TRIM54-17417_1_9 |
| SLC13A3-14820_1_2 |
| NTM-1417_5_7 |
| JSRP1-10195_4_8 |
| CNTFR-26032_1_6 |
| ZNF470-11632_1_3 |
| ZFYVE28-19650_2_7 |
| --14030_2_1 |
| OTUD7A-5252_3_7 |
| NIPAL4-20654_1_8 |
| --4772_1_1 |
| FGF11-8465_1_2 |
| SHISA2-4062_2_5 |
| EN1-16445_1_9 |
| BMP4-4774_5_4 |
| NKX2-2-14482_7_1 |
| PHOX2A-2281_1_7 |
| BHLHE23-15053_3_4 |
| SLC13A3-14820_1_5 |
| PCDHA1-20451_1_7 |
| KLHL31-22463_2_4 |
| ROBO3-1344_2_6 |
| ROBO3-1344_2_7 |
| --6882_1_8 |
| BHLHA9-7350_1_3 |
| GABRG3-5195_1_8 |
| CRELD1-19129_2_1 |
| TCERG1L-355_1_4 |
| --25442_2_3 |
| --24927_2_4 |
| --25442_2_2 |
| --24927_2_3 |
| FAM19A1-19037_1_5 |
| CECR6-15598_2_9 |
| ARTN-13933_4_2 |
| ARTN-13933_4_3 |
| ARTN-13933_4_1 |
| 44083-8547_3_5 |
| HCG11-21993_1_5 |
| 44083-8547_3_4 |
| 44083-8547_3_3 |
| 44083-8547_3_2 |
| 44083-8547_3_1 |
| CHRNB1-8467_1_3 |
| CHRNB1-8467_1_2 |
| --4829_1_5 |
| CCND1-2229_1_2 |
| CCND1-2229_1_1 |
| FLI1-1379_1_7 |
| CPPED1-5963_1_1 |
| AFAP1L2-191_1_5 |
| AFAP1L2-191_1_4 |
| AFAP1L2-191_1_3 |
| AFAP1L2-191_1_2 |
| AFAP1L2-191_1_1 |
| AKAP12-21697_1_3 |
| --14884_1_1 |
| DRD5-20146_1_6 |
| VGLL3-19083_1_1 |
| NKX2-2-14482_9_4 |
| NRG1-24833_1_10 |
| NPY5R-19469_2_6 |
| --22950_2_5 |
| EBF3-342_11_8 |
| --695_1_7 |
| JSRP1-10195_1_2 |
| --14330_3_4 |
| ADARB2-558_1_7 |
| ZNF71-11633_1_6 |
| --24444_1_9 |
| KLHDC7B-16275_2_3 |
| MOB2-1509_1_9 |
| MOB2-1509_1_8 |
| PRDM16-13628_4_1 |
| OTUD7A-5252_2_7 |
| IL4I1-11296_1_4 |
| JSRP1-10195_1_3 |
| NETO1-9264_2_2 |
| VMO1-8098_1_4 |
| REREP3-5154_4_5 |
| DRAXIN-12177_1_2 |
| FRMD4A-526_1_10 |
| ZNF470-11632_1_1 |
| SLC5A10-7485_2_4 |
| ASIC2-7677_2_6 |
| --21903_5_2 |
| MIR3687-15570_1_6 |
| TMEM151B-22413_2_8 |
| --18515_3_4 |
| --17904_2_3 |
| MMP25-6385_4_2 |
| --9199_1_9 |
| UNCX-22826_11_3 |
| UNCX-22826_11_4 |
| TFAP2A-21416_1_2 |
| --27104_1_4 |
| PARP6-5592_2_3 |
| RUNX1T1-25155_1_3 |
| PDXK-15414_1_4 |
| MAF1-24592_1_7 |
| ARHGAP35-11081_1_4 |
| PDX1-4078_1_2 |
| MOB2-1481_1_2 |
| SVEP1-25298_1_8 |
| --13327_1_9 |
| --13327_1_8 |
| HLA-F-AS1-22077_1_2 |
| RBM24-21921_1_7 |
| TXLNG-26811_3_1 |
| SPHK1-8514_9_1 |
| --26928_3_10 |
| NTM-1417_3_6 |
| SLC6A11-18007_2_6 |
| TERT-20305_3_4 |
| STARD8-27170_1_4 |
| CHST3-931_2_5 |
| RNF220-13943_1_10 |
| --17887_1_8 |
| IRS2-3760_7_2 |
| NRTN-11688_1_8 |
| FGF11-8465_1_1 |
| WDR17-19528_1_7 |
| ZYG11A-14050_3_4 |
| BCOR-26967_1_3 |
| EHMT1-25908_1_8 |
| TMEM151B-22413_2_10 |
| --25865_2_3 |
| TEX26-AS1-4114_2_7 |
| CCDC160-26598_1_9 |
| AGTR1-18338_1_3 |
| EFTUD1P1-5736_1_4 |
| IGSF9B-1430_1_7 |
| CHST8-10396_1_4 |
| TCTEX1D1-14185_1_7 |
| --26651_2_8 |
| --26575_1_9 |
| NETO1-9264_2_3 |
| SYT14-13051_2_5 |
| SALL3-9341_2_5 |
| EPCAM-17578_2_1 |
| ADAMTS2-20887_2_5 |
| --2507_3_3 |
| JSRP1-10195_1_1 |
| FAM19A1-19037_1_4 |
| --21796_3_4 |
| ZNF423-6611_2_8 |
| --26885_1_9 |
| KIF21B-12931_2_1 |
| --4772_1_3 |
| AGAP3-23055_1_5 |
| EFTUD1P1-5736_1_6 |
| OBSCN-13274_2_3 |
| OBSCN-13274_2_2 |
| OBSCN-13274_2_1 |
| --8732_1_9 |
| ADAM32-24869_1_2 |
| --14651_1_2 |
| --14651_1_1 |
| SLC6A11-18007_2_4 |
| NTSR2-16430_1_4 |
| SLC8A3-4903_4_1 |
| PRDM5-19247_1_1 |
| PCDHA1-20453_1_3 |
| --17373_1_10 |
| MIR3687-15570_1_5 |
| ADRA1D-14749_1_8 |
| ADRB3-24853_1_7 |
| NKX6-1-20073_1_1 |
| CMBL-20171_1_5 |
| CMBL-20171_1_4 |
| CMBL-20171_1_3 |
| CMBL-20171_1_2 |
| SLC10A4-19814_2_3 |
| SLC13A3-14820_1_4 |
| CRTAC1-1135_3_4 |
| LOC254559-5777_1_8 |
| --10605_1_2 |
| EDIL3-21319_1_7 |
| IRX4-20971_9_5 |
| SMTN-15853_1_3 |
| DSCR9-15326_1_7 |
| GAD2-604_1_1 |
| SVEP1-25298_1_9 |
| --22950_2_6 |
| --4772_1_2 |
| SLC10A4-19814_2_1 |
| PER3-14238_4_4 |
| ALG13-26486_2_2 |
| PACS1-2102_2_2 |
| --7398_1_9 |
| BEND4-19788_2_6 |
| NKX2-2-14482_8_3 |
| VIPR2-23267_1_10 |
| SHROOM2-27268_5_10 |
| TMEM151B-22413_2_7 |
| CYTL1-19833_1_5 |
| PPP1R36-4858_1_3 |
| PCDHA1-20453_1_4 |
| TCTEX1D1-14185_1_6 |
| MAN1A1-21528_1_1 |
| PTH1R-18815_1_2 |
| --5674_6_2 |
| --23119_4_4 |
| DNAJC6-14172_1_3 |
| --1326_1_7 |
| TTC40-428_2_4 |
| ONECUT3-9949_2_6 |
| CD99L2-26685_4_6 |
| VPS16-14542_3_2 |
| SLC13A3-14820_1_3 |
| CD34-13034_3_8 |
| CD34-13034_3_9 |
| --22327_2_6 |
| PTH2R-16968_2_5 |
| POU3F2-22623_4_7 |
| --16956_2_9 |
| KBTBD11-24705_1_7 |
| NKX2-2-14482_1_3 |
| --26641_2_9 |
| FAM110B-24975_1_1 |
| --19330_4_4 |
| LRRC71-12572_1_2 |
| SHROOM2-27268_3_6 |
| SHROOM2-27268_3_7 |
| SLC6A11-18007_2_3 |
| C1QL2-16453_3_10 |
| SHD-10862_1_2 |
| --3637_2_4 |
| FGF4-2238_1_1 |
| LINC00667-9188_1_7 |
| FAM78B-12685_1_1 |
| NRTN-11688_1_9 |
| FAM20A-8364_1_3 |
| --22333_1_3 |
| FAM43A-18584_2_7 |
| CELSR3-18847_2_8 |
| --10605_1_4 |
| KLHL1-4250_1_4 |
| BRSK2-1466_2_4 |
| IRS2-3760_7_3 |
| CNTFR-26032_1_7 |
| LOC100505876-17496_1_2 |
| LOC100505876-17496_1_1 |
| NKX2-2-14482_8_4 |
| DPY19L2P1-23523_1_8 |
| PDE1C-23503_3_5 |
| AFG3L1P-7299_2_2 |
| CDH8-6738_3_7 |
| CDH8-6738_3_8 |
| SKOR1-5557_4_1 |
| NDN-5168_1_2 |
| --15131_3_5 |
| LINGO3-10207_3_8 |
| KIF19-8418_1_1 |
| ARMCX4-26426_1_7 |
| --14264_1_6 |
| BHLHA9-7350_1_2 |
| MOB2-1506_2_6 |
| LPHN2-14252_3_1 |
| EMD-26757_7_3 |
| POU4F1-AS1-4269_1_1 |
| --6474_2_7 |
| ZNF529-10551_4_2 |
| --26641_1_6 |
| --20096_3_8 |
| --20096_3_9 |
| RIMS2-24239_1_4 |
| MTSS1-24353_2_1 |
| SALL3-9341_2_8 |
| LINC00577-21425_3_6 |
| CHRNA10-1698_1_10 |
| PARP6-5592_3_3 |
| PARP6-5592_3_2 |
| PARP6-5592_3_1 |
| --324_1_7 |
| ANKRD65-12248_3_1 |
| ZYG11A-14050_3_2 |
| JAKMIP3-375_5_3 |
| NKX6-1-20073_1_2 |
| --924_2_5 |
| --924_2_6 |
| RHOU-13286_26_1 |
| --23006_1_8 |
| --9017_3_5 |
| --24290_2_5 |
| DPP6-23105_2_3 |
| DPP6-23105_2_2 |
| RAI1-7444_1_1 |
| PTCHD1-26858_2_7 |
| LOC100287944-2557_2_5 |
| DSCR9-15326_1_8 |
| --4830_1_7 |
| LOC646862-11730_1_3 |
| GATA6-9029_1_7 |
| ZFPM2-24253_1_4 |
| ZFPM2-24253_1_3 |
| SCRT2-15193_1_4 |
| ZNF503-AS2-977_17_10 |
| --5079_1_6 |
| ZNF599-10429_1_1 |
| KIAA1462-632_2_3 |
| INSM1-14470_2_2 |
| --17094_1_1 |
| ECE2-18499_1_5 |
| LHX1-7730_3_8 |
| CPSF3-17915_2_4 |
| EFTUD1P1-5736_1_5 |
| ITPKA-5336_2_8 |
| PCDHGA1-20511_1_9 |
| FLJ42709-21352_1_1 |
| ATP8A2-4059_1_8 |
| USP35-2373_1_10 |
| IGSF9B-1430_3_3 |
| TEX28-26749_1_1 |
| NKX2-2-14482_8_5 |
| ONECUT3-9949_2_1 |
| TMEM47-26919_2_7 |
| --23119_4_5 |
| LINC00577-21425_1_9 |
| LINC00577-21425_1_10 |
| SPON1-1454_1_7 |
| --2648_1_5 |
| SVEP1-25298_1_10 |
| ONECUT3-9949_2_3 |
| GPR143-27267_1_8 |
| CLYBL-3727_2_6 |
| OBSCN-13274_2_5 |
| OBSCN-13274_2_4 |
| DPP6-23105_2_4 |
| NKX2-2-14482_4_1 |
| --4403_2_6 |
| VGLL3-19083_1_3 |
| --5861_1_1 |
| --10605_1_5 |
| ZNF512B-15165_2_1 |
| STX16-NPEPL1-14930_2_1 |
| ADRB1-185_1_3 |
| --21788_2_9 |
| AGAP1-17180_1_6 |
| LINC00577-21425_1_5 |
| --9281_1_1 |
| SLC10A3-26773_1_6 |
| DLX3-8140_2_3 |
| PLEKHH3-7883_1_4 |
| PCDHA1-20476_3_4 |
| EPAS1-17567_1_1 |
| LOC642366-21110_2_8 |
| JAKMIP3-375_2_1 |
| PRDM16-13628_11_5 |
| TTC40-425_1_9 |
| MIR548AO-21354_5_10 |
| PTH1R-18815_1_1 |
| --16685_1_7 |
| PRSS56-17151_4_1 |
| MIR4683-665_1_9 |
| MIR4683-665_1_8 |
| FLJ42875-13626_2_2 |
| --11017_1_10 |
| C1orf61-12547_2_3 |
| EBF2-24785_1_3 |
| --19546_3_1 |
| --14327_3_3 |
| --21796_2_3 |
| TRIM58-13485_2_7 |
| VGLL3-19083_1_2 |
| HES5-13456_2_1 |
| CCDC36-18865_2_4 |
| OLFM1-25687_2_4 |
| LOC100505876-17496_1_3 |
| LOC100505876-17496_1_4 |
| ADRA1A-24794_3_3 |
| PCDHGA1-20521_1_1 |
| --16102_1_4 |
| PPAP2C-10294_3_3 |
| --22251_2_10 |
| --22104_1_1 |
| LOC100288524-23509_2_6 |
| KCNN1-9996_1_4 |
| HMGA2-3557_3_2 |
| HMGA2-3557_3_1 |
| NAE1-6759_1_4 |
| CAMTA1-14229_3_1 |
| MACROD1-1969_1_9 |
| --16477_7_2 |
| ZYG11A-14050_3_3 |
| INSM1-14470_2_1 |
| --10209_1_8 |
| --4398_2_4 |
| MACROD1-1969_1_10 |
| TRIM58-13485_2_8 |
| FHL1-26624_1_5 |
| FAM26F-21510_1_1 |
| LHX3-25735_1_8 |
| PTBP1-11888_3_6 |
| --4216_2_2 |
| KLHL31-22463_1_5 |
| KLHL31-22463_1_4 |
| KLHL31-22463_1_3 |
| ACTN2-13370_1_1 |
| PCDHGA1-20511_1_10 |
| --26651_2_6 |
| SBNO2-9551_1_8 |
| --16308_2_1 |
| PCDHGA1-20521_1_4 |
| UNCX-22826_10_1 |
| EBP-27033_1_5 |
| IRX1-21034_3_9 |
| PRR23C-18276_1_7 |
| MIR3687-15570_1_4 |
| LINC00467-13067_2_6 |
| RASD1-7439_3_4 |
| LINC00693-18677_1_7 |
| LINC00693-18677_1_8 |
| PTPRN2-23195_3_7 |
| ADAM32-24869_1_7 |
| SKOR1-5557_4_2 |
| CD99L2-26685_4_7 |
| IQSEC2-27116_1_8 |
| PCDH8-4239_2_8 |
| RBFOX1-6745_2_6 |
| VSTM4-787_1_6 |
| FAM53A-19484_1_7 |
| --12331_1_2 |
| AJAP1-13991_5_1 |
| --22586_1_9 |
| SFRP1-24872_1_2 |
| BMP4-4774_5_3 |
| LRIT1-1024_1_5 |
| ASIC2-7677_4_3 |
| ROBO3-1344_1_5 |
| COX6A2-6412_2_2 |
| LINC00467-13067_1_9 |
| DBC1-25361_2_10 |
| --27045_3_9 |
| PODXL2-18144_1_5 |
| --13327_1_3 |
| PRR23C-18276_1_9 |
| PRR23C-18276_1_8 |
| SCARF2-15684_4_4 |
| LINC00605-4401_1_2 |
| FBRSL1-3018_3_8 |
| FBRSL1-3018_3_9 |
| FBRSL1-3018_3_7 |
| FBRSL1-3018_3_6 |
| AJAP1-13991_7_1 |
| SEZ6-7614_1_1 |
| RIMS2-24239_2_2 |
| --158_1_6 |
| --158_1_5 |
| --26518_2_10 |
| POU3F2-22623_3_3 |
| TONSL-24620_1_5 |
| --19784_4_9 |
| NKX2-2-14482_8_6 |
| SLC25A33-14381_2_2 |
| C14orf80-4521_1_5 |
| EDIL3-21319_2_8 |
| BHLHA9-7350_1_1 |
| TEAD3-22258_2_6 |
| --2507_3_1 |
| CD99L2-26685_4_4 |
| PODXL2-18144_5_6 |
| GPR123-447_3_5 |
| NHS-26822_1_6 |
| --14030_2_2 |
| --18108_2_3 |
| NETO1-9264_6_2 |
| HS3ST3B1-7380_1_6 |
| --19546_3_2 |
| --23126_3_1 |
| GFRA2-24723_2_8 |
| AJAP1-13991_7_2 |
| --8336_1_2 |
| RBM24-21921_1_8 |
| CD99L2-26685_3_9 |
| ALG13-26486_1_3 |
| EBF3-342_6_1 |
| MIR4683-665_1_10 |
| --15131_3_3 |
| NR2F2-5847_1_6 |
| PPAP2C-10294_6_2 |
| CELF4-9107_2_4 |
| ALDH1A3-5116_3_9 |
| SH3KBP1-26838_4_9 |
| BHLHE23-15053_3_6 |
| WDR17-19528_1_8 |
| SALL3-9341_5_3 |
| NKX2-2-14482_1_8 |
| --23391_2_1 |
| --25734_4_3 |
| SOGA3-21555_1_2 |
| TFAP2C-14899_1_2 |
| LOC642366-21110_2_5 |
| HTRA1-268_4_2 |
| HTRA1-268_4_1 |
| AMER2-4056_4_1 |
| --26641_1_5 |
| CCDC67-2462_2_1 |
| SLC30A3-17416_6_1 |
| MESP1-5792_1_3 |
| ZFPM1-7156_1_4 |
| ZFPM1-7156_1_3 |
| RASD1-7439_3_3 |
| SMOC2-21853_1_5 |
| NIPAL4-20654_1_9 |
| TMEM151B-22413_2_9 |
| --20223_1_4 |
| ZFP64-14876_1_8 |
| IRF4-22309_1_9 |
| NPY2R-19436_2_8 |
| GATA6-9029_8_7 |
| GATA6-9029_8_8 |
| GATA6-9029_8_9 |
| HS3ST6-6092_2_6 |
| LPPR3-11892_6_10 |
| PRDM5-19247_1_3 |
| SPIB-11333_1_3 |
| --22490_1_5 |
| PCDHGA1-20508_1_3 |
| LOC100133669-24487_1_1 |
| --21883_2_8 |
| --13281_1_4 |
| AGO2-24419_2_1 |
| FAM83H-24546_1_8 |
| --4440_1_5 |
| PDE1C-23503_3_4 |
| IL4I1-11296_1_5 |
| TDRD12-10369_1_5 |
| --17373_1_6 |
| --23119_2_5 |
| ATP8A2-4059_1_7 |
| AMN-4390_4_5 |
| LINGO2-25991_1_1 |
| SHD-10862_1_3 |
| CHST3-931_2_6 |
| EBF3-342_4_4 |
| LOC286177-24973_1_4 |
| EMD-26757_9_6 |
| CCBE1-9224_1_3 |
| LINGO3-10207_1_6 |
| LINGO3-10207_1_7 |
| --23782_2_9 |
| ITPKA-5336_2_10 |
| CAPN2-13183_3_6 |
| IQSEC2-27116_1_9 |
| OLIG1-15284_2_1 |
| RORB-26247_1_6 |
| --4403_2_5 |
| PACS1-2102_3_4 |
| PACS1-2102_3_5 |
| PACS1-2102_3_3 |
| PACS1-2102_3_2 |
| PACS1-2102_3_1 |
| SH3KBP1-26838_4_7 |
| TM6SF1-5730_1_3 |
| BTNL9-20943_2_5 |
| ERICH1-25001_1_6 |
| ZSCAN1-11704_2_3 |
| --21349_1_1 |
| POU3F2-22623_2_8 |
| --16982_1_7 |
| MME-18372_2_6 |
| TSPAN4-2407_4_2 |
| NKX2-2-14482_8_7 |
| --23058_2_1 |
| --5215_1_7 |
| CDH4-14958_3_8 |
| BHLHE23-15053_3_5 |
| ZNRD1-AS1-22090_1_1 |
| --14483_2_2 |
| HES5-13456_2_2 |
| --25734_2_7 |
| --21788_2_8 |
| NPBWR1-24940_2_2 |
| CSE1L-14838_1_1 |
| --9111_1_5 |
| ARMCX4-26426_1_6 |
| --3221_2_1 |
| SH3D21-13792_1_2 |
| REREP3-5154_3_3 |
| CLCN4-26431_1_4 |
| --23145_1_1 |
| --23119_3_7 |
| --20649_2_2 |
| EPHX4-14343_2_1 |
| ZNF599-10429_1_2 |
| USP35-2373_1_6 |
| --78_4_2 |
| TRIM54-17417_3_9 |
| AJAP1-13991_7_3 |
| PHACTR1-21551_1_6 |
| --22251_2_8 |
| IDUA-20154_2_4 |
| TRABD-16234_1_9 |
| NTM-1417_5_8 |
| TINCR-11536_1_1 |
| SLC6A11-18007_2_2 |
| KBTBD11-24705_5_1 |
| LOC389895-26652_1_10 |
| PRDM16-13628_7_10 |
| PHOX2A-2281_1_6 |
| GPR126-21638_1_1 |
| TXNRD2-15657_4_3 |
| TXNRD2-15657_4_2 |
| TXNRD2-15657_4_1 |
| NPTX2-24164_3_2 |
| SMC1A-27117_5_2 |
| --23782_2_10 |
| --4775_2_3 |
| GRID1-1028_4_2 |
| --1732_4_2 |
| HMX2-280_3_1 |
| FGF13-26648_2_10 |
| NTSR2-16430_1_5 |
| TBX2-8267_7_2 |
| TBX2-8267_7_1 |
| --11136_1_4 |
| PCDHGA1-20530_2_3 |
| --8827_3_7 |
| ZFPM2-24253_1_5 |
| --21883_2_7 |
| SLC22A18-1616_2_8 |
| RAPGEF4-16752_2_1 |
| IHH-17036_1_4 |
| --4398_1_2 |
| --21236_1_7 |
| IRS2-3760_2_2 |
| FAM19A5-16160_4_1 |
| --4775_2_4 |
| ZNF529-10551_5_4 |
| NKX2-2-14482_9_1 |
| --19381_1_5 |
| SALL3-9341_2_9 |
| SLC35F4-4803_2_2 |
| SALL3-9341_2_10 |
| SOX18-15172_1_4 |
| LOC100505876-17496_1_5 |
| OLFM1-25688_2_5 |
| REREP3-5154_3_2 |
| --11136_1_3 |
| NTNG2-25606_2_4 |
| --1732_3_1 |
| SNX22-5506_1_1 |
| MMP25-6385_1_1 |
| --14327_2_5 |
| PRDM16-13628_11_4 |
| RAB36-15740_1_2 |
| PXDN-16755_4_2 |
| USP35-2373_1_7 |
| SLC25A2-20497_1_5 |
| SYN2-18063_3_5 |
| PPP1R36-4858_1_2 |
| PTPRN2-23195_18_9 |
| GPC4-26594_1_1 |
| NRTN-11688_1_2 |
| LOC100288524-23509_2_4 |
| SNX20-6623_1_9 |
| FGF11-8465_1_3 |
| --2507_3_2 |
| HS3ST6-6092_2_7 |
| NKX6-1-20073_1_3 |
| EPCAM-17578_2_2 |
| HES5-13456_2_3 |
| MPO-8231_1_2 |
| ATP6V1C2-16352_3_1 |
| CLYBL-3727_1_6 |
| CLYBL-3727_1_5 |
| --24466_4_8 |
| --23119_5_2 |
| EBF3-342_6_2 |
| NKX2-2-14482_7_8 |
| LPPR2-9570_1_6 |
| --4440_1_7 |
| POU3F2-22623_4_8 |
| KBTBD11-24705_5_2 |
| NR0B1-26896_1_6 |
| FLJ42875-13626_3_4 |
| C17orf51-7540_1_4 |
| LOC100288524-23509_2_8 |
| ADAMTS2-20887_2_10 |
| ADAMTS2-20887_2_9 |
| EDIL3-21319_1_9 |
| EDIL3-21319_1_8 |
| --4395_3_3 |
| --27045_3_8 |
| AMER2-4056_4_2 |
| NPTX2-24164_3_3 |
| --10605_1_1 |
| --23145_1_4 |
| GAL3ST3-2099_2_8 |
| --15316_1_2 |
| --15316_1_3 |
| --2568_2_7 |
| WNT7B-16107_1_3 |
| --26201_1_1 |
| --26201_1_2 |
| DPP6-23105_2_1 |
| ZNF580-11591_1_2 |
| --7136_2_1 |
| --11136_1_5 |
| LOC100190940-2884_2_2 |
| PCDHA1-20453_1_1 |
| RBBP7-26812_1_7 |
| IGSF9B-1430_1_9 |
| --8732_1_10 |
| CTNND2-20225_4_3 |
| --25097_2_5 |
| --25097_2_4 |
| FAM43A-18584_2_5 |
| --24649_1_7 |
| MAMLD1-26681_2_8 |
| PRDM16-13628_8_5 |
| KBTBD11-24705_5_3 |
| AJAP1-13991_7_4 |
| TMEFF2-16873_1_7 |
| CRTAC1-1135_3_3 |
| C2orf40-16340_1_3 |
| NRTN-11688_1_1 |
| URGCP-23584_1_2 |
| URGCP-23584_1_1 |
| CPLX1-20012_1_3 |
| --14651_1_3 |
| GRID1-1028_4_3 |
| --9141_1_9 |
| NPY2R-19436_2_9 |
| SCRT2-15193_1_1 |
| HOXD-AS2-16795_1_1 |
| CSE1L-14838_1_2 |
| POU4F1-AS1-4269_1_2 |
| --16102_1_5 |
| --9012_2_7 |
| --4829_3_2 |
| LOC101055625-3132_1_7 |
| UNCX-22826_3_1 |
| MIR3687-15570_3_7 |
| GPC4-26594_2_4 |
| --23058_1_7 |
| ZNF536-10330_1_3 |
| PAPLN-4918_1_1 |
| BMP4-4774_1_1 |
| HTRA1-268_4_3 |
| HTRA1-268_4_4 |
| HTRA1-268_4_5 |
| HTRA1-268_4_6 |
| --9340_1_7 |
| DAW1-17105_3_10 |
| --9141_1_10 |
| RHOU-13286_14_2 |
| --4405_3_8 |
| QRFPR-19252_2_7 |
| GRM4-22231_4_1 |
| SIM2-15317_3_3 |
| SIM2-15317_3_2 |
| SLC8A3-4903_4_2 |
| CLCN4-26431_1_3 |
| ADARB2-558_1_3 |
| LOC100288524-23509_2_5 |
| ITGA11-5567_1_3 |
| NKX2-2-14482_9_5 |
| --17283_4_7 |
| DPP6-23105_2_5 |
| PRDM16-13628_11_9 |
| SLC6A11-18007_1_1 |
| MINK1-8136_5_5 |
| CNTN4-18651_4_2 |
| TMEM132E-7681_1_7 |
| ABCB1-24079_2_8 |
| --78_4_4 |
| ARMCX4-26426_1_5 |
| IKZF1-23670_5_1 |
| CNTN4-18651_4_1 |
| ALDH1A2-5462_2_7 |
| CD99L2-26685_4_3 |
| MPPED1-16070_2_4 |
| NEURL-136_2_6 |
| --13481_1_6 |
| NHS-26821_1_5 |
| --4216_2_1 |
| ELOVL2-21480_3_1 |
| BAIAP2-8692_4_3 |
| BAIAP2-8692_4_2 |
| ABCB1-24079_2_4 |
| ADARB2-558_1_2 |
| --21238_1_6 |
| --21238_1_7 |
| KBTBD11-24705_2_1 |
| IGSF9B-1430_1_8 |
| GRID1-1028_4_4 |
| MIR3687-15570_3_6 |
| BHLHA9-7350_1_8 |
| LHX5-2647_2_4 |
| PHACTR1-21551_1_7 |
| CCDC67-2462_2_2 |
| --22071_2_8 |
| --3058_2_1 |
| LAYN-1198_1_3 |
| HHEX-1076_1_2 |
| ADRA1D-14749_1_6 |
| FLJ42875-13626_4_7 |
| BMP4-4774_1_2 |
| --23525_3_7 |
| --7398_1_8 |
| --7398_1_7 |
| --7398_1_6 |
| --7398_1_5 |
| --7398_1_4 |
| --7398_1_3 |
| AMER2-4056_3_3 |
| BARHL2-14324_1_5 |
| NETO1-9264_6_1 |
| VAT1L-6950_2_3 |
| EFTUD1P1-5736_1_7 |
| PPAP2C-10294_3_9 |
| ATP2A3-7830_1_1 |
| --21614_2_7 |
| LMF1-5883_6_5 |
| HOXD-AS1-16808_3_7 |
| LPPR2-9570_1_7 |
| --9238_1_7 |
| --6835_2_3 |
| REREP3-5154_4_3 |
| --11717_1_8 |
| --24366_3_5 |
| --24366_3_4 |
| --24366_3_3 |
| NKX2-4-14481_6_8 |
| CLDN6-6363_1_4 |
| SMC1A-27117_4_3 |
| CPLX1-20003_2_7 |
| VENTX-476_1_1 |
| PRDM5-19247_1_4 |
| --18155_2_6 |
| LINC00261-14491_1_1 |
| HMX1-20101_1_5 |
| ADCYAP1R1-23499_4_3 |
| --19381_2_5 |
| SOX10-15931_1_5 |
| --20238_3_5 |
| PPAP2C-10294_6_5 |
| IRX1-21034_3_8 |
| EBF3-342_4_5 |
| --13481_1_5 |
| PTPRN2-23195_18_10 |
| --5674_6_3 |
| --26402_1_7 |
| IRF8-7044_4_8 |
| OTX2-4797_1_1 |
| BTNL9-20943_2_6 |
| BTBD19-13956_1_10 |
| AMER2-4056_3_4 |
| ZNF580-11591_1_1 |
| RBP4-1091_7_1 |
| ST8SIA3-9198_1_6 |
| NOL4-9078_1_1 |
| BAHCC1-8721_2_2 |
| EN1-16444_2_1 |
| M1AP-17791_1_7 |
| --7136_2_2 |
| PCDHA1-20472_1_3 |
| CTNND2-20250_2_5 |
| CTNND2-20250_2_6 |
| TBX2-8267_4_9 |
| SHISA9-5964_1_3 |
| SHISA9-5964_1_2 |
| HOXD-AS2-16795_1_2 |
| --14327_3_5 |
| LINGO3-10207_3_6 |
| BEND4-19788_2_1 |
| TMEM136-1299_2_1 |
| --7218_1_2 |
| OTX2-4797_1_2 |
| ALDH1A2-5462_2_10 |
| PLK3-13955_1_9 |
| GPC4-26594_2_10 |
| PLLP-6693_1_2 |
| --6835_1_3 |
| RGS17-21711_3_3 |
| --10404_1_6 |
| --10404_1_5 |
| RIMS2-24239_1_5 |
| --3637_1_2 |
| CHST3-931_1_7 |
| FBXO43-24208_1_2 |
| --4184_1_8 |
| --21754_1_5 |
| MXRA8-12242_6_10 |
| RUNX3-13503_6_3 |
| POU3F2-22623_5_5 |
| PHOX2A-2281_1_4 |
| ADAMTS2-20887_2_6 |
| --9238_3_4 |
| --9012_2_6 |
| VAC14-6872_5_1 |
| --3058_2_3 |
| ALK-17444_1_1 |
| --21788_2_7 |
| SPIB-11333_1_4 |
| --3202_1_1 |
| --3202_1_2 |
| ANKRD65-12248_2_1 |
| ZNF599-10429_1_3 |
| ZNF599-10429_1_4 |
| CCDC166-24543_1_4 |
| --25865_2_1 |
| SBK2-11578_1_4 |
| SOX10-15931_1_7 |
| LINC00466-14145_3_5 |
| RELN-22720_1_5 |
| --2507_4_7 |
| --4398_1_4 |
| --4398_1_3 |
| OLFM1-25688_2_6 |
| ACTN2-13370_1_2 |
| MVD-7163_1_7 |
| --14651_1_4 |
| INPP5A-409_5_5 |
| --19546_3_3 |
| STK3-25209_2_3 |
| --20223_1_6 |
| MOB2-1481_1_1 |
| CTNND2-20225_4_1 |
| --23754_1_1 |
| CYP2F1-10802_1_6 |
| CYP2F1-10802_1_7 |
| C9orf172-25809_7_8 |
| --8735_1_9 |
| --25369_1_4 |
| CCDC67-2462_2_3 |
| REREP3-5154_6_4 |
| PTPRU-13624_3_3 |
| --6479_1_6 |
| AMER2-4056_4_3 |
| FUT4-2476_1_7 |
| EBF3-341_2_2 |
| KCNN1-9996_1_5 |
| --3388_1_2 |
| TRIM15-22099_1_4 |
| MINK1-8136_5_4 |
| --1732_4_1 |
| FLJ42875-13626_2_1 |
| LOC100288524-23509_3_9 |
| RUNDC3B-24081_1_1 |
| AMER2-4056_5_7 |
| CCDC85A-17619_1_1 |
| SERP2-4179_4_8 |
| SERP2-4179_4_7 |
| --22490_1_4 |
| GPR137B-13363_6_5 |
| GPR137B-13363_6_4 |
| GPR137B-13363_6_3 |
| LINC00577-21425_1_6 |
| ANKRD65-12248_3_2 |
| PPP1R13L-10984_3_3 |
| SCARF2-15684_2_10 |
| CTNND2-20225_4_2 |
| GALNT18-1231_1_1 |
| ADAMTS2-20887_2_3 |
| --9012_2_5 |
| POU4F1-AS1-4269_1_3 |
| SLC43A1-1843_1_1 |
| ST8SIA5-9129_1_10 |
| --13481_1_7 |
| PIM2-27048_1_4 |
| --22251_2_7 |
| LINC00629-26603_1_7 |
| PIGZ-18626_1_5 |
| BMPR1B-20138_1_1 |
| ZNF423-6611_1_1 |
| --26365_4_5 |
| PTPRT-14743_1_3 |
| SHISA9-5964_1_5 |
| SHISA9-5964_1_4 |
| FAM132A-12185_1_7 |
| EFCAB4B-3176_2_3 |
| PNCK-26727_1_6 |
| RIMS2-24239_1_1 |
| TRPS1-24302_1_3 |
| --4052_1_3 |
| --15511_2_1 |
| EPAS1-17567_1_2 |
| FOXP2-22775_4_4 |
| FOXP2-22775_4_5 |
| --4971_2_3 |
| --4440_1_8 |
| NPTX2-24164_2_4 |
| SALL3-9341_5_6 |
| SALL3-9341_5_5 |
| SALL3-9341_5_7 |
| --23145_1_2 |
| --3221_2_2 |
| AMPH-23546_1_2 |
| SPIB-11333_1_2 |
| INPP5A-409_5_4 |
| --17521_14_8 |
| LINC00467-13067_1_10 |
| BMP3-20039_3_10 |
| LOC100506421-16317_3_7 |
| PTER-549_1_2 |
| RASIP1-11185_3_8 |
| ROR1-14149_1_3 |
| ROR1-14149_1_2 |
| ROR1-14149_1_1 |
| TWIST2-17229_2_5 |
| --17141_2_9 |
| --17141_2_8 |
| BHLHE23-15053_3_2 |
| NEURL-136_2_7 |
| PDE4B-14177_2_1 |
| --16622_1_1 |
| PKDREJ-16120_3_10 |
| LINGO3-10207_3_4 |
| ASIC2-7677_2_7 |
| GDF6-25183_5_2 |
| --26201_1_3 |
| GDF6-25183_5_1 |
| C1QL3-550_2_8 |
| PTCHD1-26858_2_8 |
| UBE2T-12952_1_3 |
| --9280_1_5 |
| --17725_1_4 |
| --17725_1_5 |
| --17725_1_3 |
| SYN2-18063_1_2 |
| KBTBD11-24705_1_6 |
| --18155_2_5 |
| DUPD1-972_1_4 |
| --14483_2_3 |
| FLJ42709-21351_4_9 |
| RELN-22720_1_6 |
| --7218_1_3 |
| NTM-1417_4_1 |
| --16723_1_2 |
| --21796_2_4 |
| PTPRM-9443_1_1 |
| JPH3-7090_8_10 |
| NTSR2-16430_1_2 |
| CD99L2-26685_5_1 |
| TFAP2A-21416_1_3 |
| IDUA-20154_1_1 |
| IKZF1-23670_2_1 |
| CABP7-15824_3_1 |
| SQSTM1-20911_1_8 |
| SQSTM1-20911_1_7 |
| RORB-26247_1_1 |
| NETO1-9264_6_3 |
| BTNL9-20943_2_7 |
| CYP2F1-10802_1_8 |
| INPP5A-409_5_6 |
| MMP25-6385_4_3 |
| SMC1A-27117_4_1 |
| --21883_2_6 |
| --16884_2_4 |
| --16884_2_5 |
| RPS6KA3-26842_4_1 |
| LECT1-4238_4_1 |
| RHOU-13286_54_1 |
| RHOU-13286_54_2 |
| RHOU-13286_54_3 |
| NKX2-2-14482_1_4 |
| FAM78B-12685_1_2 |
| MSX1-19819_1_5 |
| ZIC2-3731_3_1 |
| CABIN1-15764_1_2 |
| CABIN1-15764_1_1 |
| --12080_2_4 |
| --12080_2_3 |
| ZNF71-11633_1_7 |
| ZNF470-11632_1_9 |
| CSDA-2572_1_3 |
| --9280_1_6 |
| --9280_1_1 |
| RNF220-13943_1_6 |
| MME-18372_1_5 |
| SHC3-26312_1_8 |
| --6479_1_5 |
| RAB9A-26643_1_2 |
| HLCS-15322_1_1 |
| RUNX1-15305_1_5 |
| THRB-18659_1_1 |
| ZNF697-12206_1_4 |
| FAM84B-24360_1_3 |
| --6835_1_4 |
| SMC1A-27117_4_4 |
| C9orf172-25809_7_3 |
| --14651_1_5 |
| HES5-13456_2_4 |
| PPP1R13L-10984_3_4 |
| NIPAL4-20654_1_10 |
| LMF1-5883_4_1 |
| MIR3687-15570_1_3 |
| FAM84B-24360_1_5 |
| --2454_2_5 |
| SMG6-7507_5_5 |
| --4241_1_7 |
| VENTX-476_1_2 |
| RUNX1-15305_1_4 |
| NDN-5168_1_6 |
| CSMD2-13742_1_4 |
| --22529_1_8 |
| ACHE-22665_5_3 |
| VWC2-23663_3_6 |
| IDUA-20154_2_3 |
| UQCRH-13977_1_1 |
| SFRP1-24872_1_3 |
| --5441_2_5 |
| UNCX-22826_11_6 |
| LOC100288524-23509_2_10 |
| ONECUT1-5437_2_4 |
| LOC100288524-23509_2_9 |
| TLX2-17787_4_6 |
| ATP8B3-9979_1_1 |
| IQSEC1-18205_2_10 |
| NTM-1417_3_7 |
| AJAP1-13991_7_5 |
| PHOX2A-2281_1_5 |
| CACNA2D4-3079_2_7 |
| --2648_1_7 |
| --2648_1_6 |
| VSTM2A-23715_1_1 |
| GPC4-26594_3_3 |
| UNCX-22826_11_2 |
| FLJ42875-13626_2_3 |
| NKX2-4-14481_6_9 |
| NKX2-4-14481_6_10 |
| ADARB2-558_1_4 |
| IRX4-20971_9_6 |
| CTNND2-20225_4_5 |
| TMTC1-3135_2_2 |
| ST8SIA5-9129_1_5 |
| --26928_2_4 |
| --17141_1_4 |
| CLDN6-6363_1_3 |
| TERT-20305_4_2 |
| PPAP2C-10294_6_3 |
| TRIM58-13485_2_6 |
| --15511_2_2 |
| LINGO3-10207_3_7 |
| FAM53A-19484_1_3 |
| --23657_2_6 |
| CASKIN1-6157_2_10 |
| --24444_1_10 |
| STX16-NPEPL1-14929_3_3 |
| LY86-AS1-22494_1_8 |
| --3221_3_8 |
| --11017_1_9 |
| LINGO3-10207_3_5 |
| INPP5A-409_5_1 |
| LMF1-5883_4_2 |
| SALL3-9341_6_5 |
| 44085-20004_1_1 |
| --725_1_2 |
| --7136_2_3 |
| NDRG4-6719_2_1 |
| NDRG4-6719_2_2 |
| ANKS1B-3720_2_3 |
| IDUA-20154_1_2 |
| --24366_3_2 |
| SLC10A3-26773_1_9 |
| SLC10A3-26773_1_10 |
| --2507_2_4 |
| --4971_2_4 |
| DEPDC7-1663_1_1 |
| PER3-14238_4_6 |
| INPP5A-409_7_1 |
| PRMT2-15564_2_1 |
| SLC39A4-24614_2_4 |
| --24366_3_1 |
| TMEM47-26919_3_6 |
| ZIC1-18332_2_8 |
| NETO1-9264_2_4 |
| NDN-5168_1_1 |
| VSTM4-787_1_7 |
| TDH-24281_2_2 |
| SCRT2-15193_2_2 |
| SHC3-26312_1_10 |
| ZFPM1-7156_1_2 |
| SCRT2-15193_1_5 |
| IRAK1-26746_1_5 |
| --506_1_8 |
| SDF4-12160_3_5 |
| GALR1-9303_1_4 |
| JSRP1-10195_4_10 |
| PACS1-2102_3_6 |
| --15795_3_1 |
| DFNB31-25353_1_3 |
| FOXG1-4658_2_6 |
| OLFM1-25687_4_4 |
| OPCML-1419_1_7 |
| --20997_1_5 |
| --9012_2_3 |
| LPL-24710_3_5 |
| PRDM16-13628_11_3 |
| BAHCC1-8721_2_4 |
| SFMBT2-944_4_6 |
| INPP5A-409_10_1 |
| --3814_1_5 |
| PLCB3-1985_1_1 |
| --14327_3_4 |
| --4216_2_5 |
| DUPD1-972_1_3 |
| DCAF12L1-26559_1_10 |
| NR2F6-9929_1_2 |
| SLC6A11-18007_2_1 |
| --4358_1_10 |
| MOB2-1481_3_9 |
| MOB2-1481_3_10 |
| SOX10-15931_1_6 |
| SERHL2-16047_2_1 |
| GPR12-4067_1_7 |
| SALL3-9341_4_9 |
| --24926_1_9 |
| LOC100131320-16539_1_6 |
| LOC100131320-16539_1_5 |
| NKD2-20161_3_8 |
| NKD2-20161_3_7 |
| FAM58A-26720_1_1 |
| JSRP1-10195_4_9 |
| HCFC1-26744_1_6 |
| --4971_1_2 |
| --9012_2_4 |
| ARMCX4-26426_1_4 |
| CNTN4-18651_4_3 |
| CNTN4-18651_4_4 |
| IRX4-20971_9_4 |
| LOC100131320-16539_1_4 |
| LOC100131320-16539_1_3 |
| TNFRSF11A-9234_3_1 |
| SLC9A3-21120_3_9 |
| --8336_1_1 |
| CLCN4-26431_1_2 |
| KIF19-8416_1_1 |
| GRID1-1028_9_3 |
| UNCX-22826_4_1 |
| POU3F2-22623_2_10 |
| POU3F2-22623_2_9 |
| --22863_1_3 |
| LMF1-5883_4_3 |
| --3058_2_2 |
| FLJ42875-13626_3_2 |
| NECAB1-25150_1_2 |
| NR5A1-25417_7_5 |
| HOXD-AS1-16807_1_2 |
| IDUA-20154_3_5 |
| ZNF71-11633_1_8 |
| FGF4-2238_1_2 |
| --5441_2_8 |
| SPIB-11333_1_1 |
| EBF3-342_7_1 |
| EBF3-342_7_2 |
| M1AP-17791_1_8 |
| SSTR4-14495_1_6 |
| LOC100131320-16539_1_2 |
| LOC100131320-16539_1_1 |
| SMOC2-21853_2_8 |
| SLC6A11-18007_3_8 |
| HS3ST3B1-7380_1_5 |
| --5111_2_6 |
| GALR1-9303_1_5 |
| TRIM2-19412_2_2 |
| FOXH1-24627_1_5 |
| ST8SIA5-9129_1_6 |
| RPS6KA3-26842_4_7 |
| C5orf38-20998_1_9 |
| C5orf38-20998_1_8 |
| AJAP1-13991_3_10 |
| PCDHA1-20472_2_7 |
| --7011_1_3 |
| --22071_2_9 |
| SFRP1-24872_2_1 |
| NEIL3-19536_1_1 |
| ARX-26874_4_8 |
| ADRA1D-14749_1_7 |
| --15797_1_4 |
| --15797_1_3 |
| PTPRN2-23193_1_9 |
| NDN-5168_1_5 |
| --17560_1_10 |
| --17141_1_5 |
| TRIM54-17417_1_8 |
| GPNMB-23370_1_9 |
| FKBP5-22261_1_2 |
| UNCX-22826_5_5 |
| FKBP5-22261_1_1 |
| --18108_1_8 |
| --18108_1_7 |
| CCDC102A-6697_2_9 |
| --823_1_7 |
| MIR3687-15570_3_5 |
| SALL3-9341_5_4 |
| --9017_3_7 |
| --5441_2_6 |
| KIF26B-13455_3_7 |
| ERICH1-25001_1_7 |
| KREMEN2-6329_3_5 |
| SMC1A-27117_4_2 |
| PRDM16-13628_11_10 |
| DLL1-21902_1_1 |
| KLF2-9895_2_5 |
| TPTEP1-15591_1_6 |
| LINGO3-10207_3_3 |
| ZFPM2-24253_1_1 |
| ARX-26874_4_7 |
| --19330_4_2 |
| --16412_1_5 |
| --16412_1_3 |
| --16412_1_4 |
| --16412_1_2 |
| --16412_1_1 |
| PTPRN2-23193_1_10 |
| CXCL12-733_1_5 |
| UNCX-22826_7_1 |
| TRIM54-17417_1_5 |
| SFRP1-24872_1_4 |
| ZNF529-10551_5_6 |
| ZNF529-10551_5_5 |
| TACR1-17798_1_1 |
| --24466_3_1 |
| --20238_3_6 |
| PDXK-15414_1_1 |
| LMF1-5883_6_6 |
| --9012_2_1 |
| HLA-F-AS1-22077_1_3 |
| SCT-1944_2_3 |
| --9199_1_10 |
| --23145_1_3 |
| ATP8B3-9979_1_2 |
| IKZF1-23670_3_2 |
| IKZF1-23670_3_1 |
| MCF2-26650_1_4 |
| SALL3-9341_2_3 |
| LOC100216546-22724_1_2 |
| LOC100216546-22724_1_1 |
| EPB41L3-9207_1_9 |
| --21754_1_6 |
| TEAD3-22258_2_4 |
| GATA3-993_4_1 |
| JAKMIP3-375_2_2 |
| UQCRH-13977_1_2 |
| TWIST1-23309_1_1 |
| RLTPR-6802_2_2 |
| IDUA-20154_2_2 |
| RLTPR-6802_2_4 |
| CRTAC1-1135_1_1 |
| GATA2-18154_3_8 |
| FOXA1-4712_2_5 |
| PTH2R-16968_2_3 |
| NRN1-22486_4_6 |
| GPR4-11006_1_3 |
| GPR4-11006_1_2 |
| C16orf58-6419_2_8 |
| OLFM1-25687_2_1 |
| EBF2-24785_5_7 |
| CACNA1H-5938_3_1 |
| ONECUT3-9952_1_7 |
| PCDHA1-20472_2_5 |
| --24270_1_8 |
| --4184_1_9 |
| TP73-13766_3_10 |
| FAM19A5-16160_3_3 |
| HOXB3-8064_2_7 |
| HOXB3-8064_2_8 |
| PER3-14238_4_5 |
| PTCHD1-26858_1_2 |
| LHX2-25407_4_5 |
| LHX2-25407_4_4 |
| LHX2-25407_4_3 |
| SLC10A4-19814_2_2 |
| CLYBL-3727_2_7 |
| --5441_2_4 |
| XKR6-24271_1_5 |
| KCNJ12-7534_3_6 |
| FAM47E-STBD1-19994_1_2 |
| FAM47E-STBD1-19994_1_1 |
| PCDHA1-20476_3_3 |
| CNTNAP5-16488_1_2 |
| --26641_1_7 |
| DACT2-21847_3_1 |
| TRABD-16234_2_9 |
| SNX20-6623_1_7 |
| --5341_1_2 |
| TRABD2B-14014_1_1 |
| PHOX2A-2281_1_3 |
| TCEA2-15175_2_1 |
| --26641_2_8 |
| FGFRL1-19147_2_7 |
| VENTX-476_1_9 |
| ABCA3-6196_7_1 |
| HMGA1-22235_9_2 |
| HMGA1-22235_9_1 |
| FAM78B-12685_2_3 |
| OLFM1-25687_4_2 |
| ZNF423-6611_2_7 |
| SHC3-26312_1_7 |
| EBF3-342_7_3 |
| EBF3-342_7_4 |
| ZNF599-10429_1_5 |
| COL18A1-15492_1_1 |
| ALDH1A3-5116_3_8 |
| EHMT1-25908_1_9 |
| EDIL3-21319_1_10 |
| SLC34A2-19668_2_3 |
| SLC34A2-19668_2_1 |
| SLC34A2-19668_2_2 |
| SALL3-9341_5_10 |
| SALL3-9341_5_8 |
| SALL3-9341_5_9 |
| ZIC2-3731_3_2 |
| --6595_1_8 |
| --17521_14_7 |
| --17521_14_6 |
| --17521_14_4 |
| --17521_14_5 |
| --17521_14_3 |
| --17521_14_2 |
| --17521_14_1 |
| --4927_1_4 |
| --13481_1_4 |
| FAM222A-2590_5_1 |
| SLC27A1-9953_1_1 |
| GPC4-26594_1_2 |
| --9340_2_9 |
| ADAMTS7-5694_2_2 |
| --23754_1_4 |
| --23058_2_3 |
| --4083_1_6 |
| --3165_1_2 |
| LOC642366-21110_2_6 |
| PTPRN2-23243_4_6 |
| ADRB3-24853_1_2 |
| SHANK3-16286_1_3 |
| LOC100132891-25058_1_5 |
| --14466_2_5 |
| OPLAH-24584_6_6 |
| SLC13A3-14820_1_1 |
| ZNF718-19176_2_3 |
| NEGR1-14213_1_2 |
| --20427_1_1 |
| GRIK3-13802_2_1 |
| 44084-16361_1_3 |
| PTPRM-9308_3_1 |
| --12331_1_1 |
| NTM-1417_4_2 |
| --10605_3_6 |
| ACTN2-13370_2_3 |
| --13836_1_4 |
| TCERG1L-355_3_7 |
| --16477_7_1 |
| FBXL21-20376_1_10 |
| GRM5-2433_3_4 |
| NETO1-9264_8_6 |
| JPH3-7090_4_10 |
| --798_2_8 |
| ZMYND10-18910_2_9 |
| NPTX1-8663_3_1 |
| BTNL9-20943_1_2 |
| NTM-1417_5_9 |
| ZMYND10-18910_2_8 |
| HELZ2-15118_1_10 |
| VPS51-2036_1_8 |
| --26885_1_8 |
| ALG13-26486_1_2 |
| GRIN3A-25260_1_8 |
| RTN4RL2-1840_4_1 |
| ITPKB-13228_3_9 |
| IRX5-6650_5_5 |
| PCDHGA1-20530_2_10 |
| KCNK17-22312_1_1 |
| ALG13-26486_1_1 |
| EBF3-342_10_1 |
| ZNF718-19176_2_4 |
| FLJ42709-21351_4_10 |
| PRKG1-813_2_5 |
| EFEMP2-2089_1_5 |
| MOB2-1506_2_7 |
| MPPED1-16070_2_6 |
| MPPED1-16070_2_7 |
| NKX2-2-14482_9_6 |
| FLJ42875-13626_2_4 |
| HEATR4-4922_2_2 |
| HEATR4-4922_2_3 |
| --20238_3_4 |
| CAV2-22782_1_2 |
| CAV2-22782_1_1 |
| GRIK3-13802_2_2 |
| SLC25A39-7950_1_2 |
| GATA6-9029_2_1 |
| PTPRN2-23243_4_1 |
| CNTFR-26032_2_8 |
| CACNA2D1-24059_2_3 |
| CABIN1-15764_1_6 |
| CACNA2D1-24059_2_1 |
| LOX-20260_1_3 |
| CACNA2D1-24059_2_2 |
| GDF6-25183_3_3 |
| CABIN1-15764_1_5 |
| GDF6-25183_3_2 |
| CABIN1-15764_1_4 |
| CABIN1-15764_1_3 |
| WNT3A-13251_2_1 |
| ATP6V0C-6212_1_1 |
| ATP6V0C-6212_1_2 |
| --761_4_3 |
| --761_4_9 |
| --761_4_4 |
| --761_4_10 |
| --761_4_6 |
| --761_4_8 |
| --761_4_5 |
| --761_4_7 |
| SHANK3-16286_1_5 |
| IRX4-20971_7_10 |
| PDX1-4078_1_3 |
| OBSCN-13262_1_9 |
| HEBP2-21619_1_1 |
| --9141_1_8 |
| BARHL2-14324_3_10 |
| BMP4-4774_5_2 |
| OLFM1-25687_4_3 |
| PHF21B-16090_5_3 |
| BAHCC1-8721_2_3 |
| CLCN4-26431_1_1 |
| OLFM1-25687_1_6 |
| GRM7-19039_2_6 |
| --924_2_4 |
| RIN3-5057_1_5 |
| VENTX-476_1_8 |
| RBM24-21921_1_6 |
| BMP7-14904_1_6 |
| BMP3-20039_3_9 |
| TRIM54-17417_2_1 |
| SPON1-1454_1_2 |
| SCRIB-24556_2_5 |
| --25734_2_8 |
| NRG3-1016_1_8 |
| ADAL-5364_1_9 |
| ARMCX4-26426_1_9 |
| OLFM1-25687_2_2 |
| TTC40-428_4_7 |
| --23145_1_6 |
| APEX2-27130_1_5 |
| APEX2-27130_1_6 |
| SCUBE1-16069_2_3 |
| CMBL-20171_2_1 |
| --23145_1_5 |
| --9212_1_1 |
| SIX2-17558_1_3 |
| --15131_3_2 |
| --5843_1_1 |
| FAM78B-12685_2_4 |
| BMP2-15195_2_2 |
| LINGO2-25991_1_6 |
| LINGO2-25991_1_4 |
| LINGO2-25991_1_5 |
| LINGO2-25991_1_2 |
| LINGO2-25991_1_3 |
| ABCA3-6196_7_5 |
| GABBR2-25234_5_6 |
| ABCA3-6196_7_3 |
| ABCA3-6196_7_2 |
| ABCA3-6196_7_4 |
| CPM-3588_5_2 |
| --4029_1_3 |
| --7159_1_8 |
| FLJ42709-21351_4_7 |
| PABPC5-27261_1_7 |
| NOL4-9077_1_1 |
| --22251_2_2 |
| UNCX-22826_3_2 |
| JSRP1-10195_4_7 |
| YDJC-15718_1_6 |
| NEIL3-19536_1_2 |
| LANCL3-26939_2_8 |
| C11orf92-1195_1_3 |
| PHOX2A-2281_1_2 |
| RLTPR-6802_2_5 |
| GPR176-5295_5_1 |
| NDN-5168_1_3 |
| ZNF248-673_1_3 |
| OTUD7A-5252_2_6 |
| --21788_1_3 |
| --21788_1_2 |
| TRIM54-17417_1_4 |
| LINC00461-21334_2_1 |
| --13327_1_2 |
| --14706_2_4 |
| --10209_2_8 |
| PTPRN2-23243_4_2 |
| MPPED1-16070_2_5 |
| CCDC160-26598_3_3 |
| SPON1-1454_1_3 |
| --22251_2_1 |
| UBL4A-26772_3_3 |
| HS3ST3B1-7380_1_7 |
| CPNE5-22280_1_7 |
| KLHDC7B-16275_2_6 |
| SLC12A5-14803_1_1 |
| IKZF1-23670_3_3 |
| --25056_1_4 |
| --78_4_1 |
| --732_1_5 |
| TLX2-17787_4_5 |
| SOX1-3813_1_5 |
| GPR137B-13363_1_1 |
| CNTNAP5-16488_1_3 |
| DNAJC6-14172_1_4 |
| KCNQ1DN-1611_1_4 |
| KCNQ1DN-1611_1_5 |
| PTGDR-4763_3_7 |
| LINGO3-10207_3_2 |
| DNER-17108_1_8 |
| IDUA-20154_2_1 |
| UBL4A-26772_3_4 |
| --10605_3_5 |
| RORB-26247_1_2 |
| LOC284801-14531_1_8 |
| SFMBT2-944_4_7 |
| GPC4-26594_2_5 |
| SMS-26851_4_5 |
| CTNND2-20225_4_4 |
| RELN-22720_1_8 |
| STX16-NPEPL1-14929_3_5 |
| STX16-NPEPL1-14929_3_6 |
| STX16-NPEPL1-14929_3_4 |
| GPR26-283_2_3 |
| UNCX-22826_5_6 |
| LHX5-2646_1_3 |
| --25734_4_4 |
| LMX1A-12673_3_4 |
| --4041_1_7 |
| RUNX1T1-25155_1_1 |
| ATP10A-5183_2_2 |
| SLC9A7-27007_4_5 |
| PDE10A-21786_2_5 |
| SERP2-4179_4_1 |
| SERP2-4179_4_2 |
| SLITRK4-26662_2_6 |
| ADARB2-558_2_1 |
| MINK1-8136_4_3 |
| --9280_1_7 |
| CNTN4-18651_2_8 |
| CRTAC1-1135_4_4 |
| ZNF804A-16845_3_9 |
| UNCX-22826_7_2 |
| S1PR2-9485_2_2 |
| LOC100131320-16539_1_8 |
| LOC100131320-16539_1_7 |
| RET-708_1_5 |
| GNAZ-15737_3_1 |
| FLJ42875-13626_2_5 |
| TONSL-24620_1_4 |
| ADRB3-24853_1_9 |
| PTH2R-16968_2_4 |
| ADRB3-24853_1_8 |
| ZNF497-11725_2_6 |
| LMF1-5883_6_7 |
| PARD6G-9441_4_2 |
| DRD4-1965_4_5 |
| STAC-18706_1_1 |
| MBNL3-26591_1_8 |
| CRTAC1-1135_3_1 |
| IRF4-22309_1_10 |
| HCFC1-26744_1_2 |
| --23058_2_2 |
| SH3KBP1-26838_5_8 |
| --14327_3_6 |
| NECAB1-25150_1_3 |
| NECAB1-25150_1_4 |
| --11113_2_5 |
| RORB-26247_1_4 |
| GAS2L1-15817_1_3 |
| ACSL4-26480_2_9 |
